# Sulfonolipids as novel metabolite markers of *Alistipes* and *Odoribacter* affected by high-fat diets

**DOI:** 10.1038/s41598-017-10369-z

**Published:** 2017-09-08

**Authors:** Alesia Walker, Barbara Pfitzner, Mourad Harir, Monika Schaubeck, Jelena Calasan, Silke S. Heinzmann, Dmitrij Turaev, Thomas Rattei, David Endesfelder, Wolfgang zu Castell, Dirk Haller, Michael Schmid, Anton Hartmann, Philippe Schmitt-Kopplin

**Affiliations:** 1 Research Unit Analytical BioGeoChemistry, Helmholtz Zentrum München, German Research Center for Environmental Health, Neuherberg, Germany; 2 Research Unit Microbe-Plant Interactions, Research Group Molecular Microbial Ecology, Helmholtz Zentrum München, German Research Center for Environmental Health, Neuherberg, Germany; 3 Chair of Nutrition and Immunology, Technische Universität München, Freising-Weihenstephan, Germany; 4 Division of Computational System Biology, Department of Microbiology and Ecosystem Science, University of Vienna, Vienna, Austria; 5 Scientific Computing Research Unit, Helmholtz Zentrum München, German Research Center for Environmental Health, Neuherberg, Germany; 6 Chair of Analytical Food Chemistry, Technische Universität München, Freising-Weihenstephan, Germany; 7 ZIEL – Institute for Food & Health, Technische Universität München, Freising-Weihenstephan, Germany

## Abstract

The gut microbiota generates a huge pool of unknown metabolites, and their identification and characterization is a key challenge in metabolomics. However, there are still gaps on the studies of gut microbiota and their chemical structures. In this investigation, an unusual class of bacterial sulfonolipids (SLs) is detected in mouse cecum, which was originally found in environmental microbes. We have performed a detailed molecular level characterization of this class of lipids by combining high-resolution mass spectrometry and liquid chromatography analysis. Eighteen SLs that differ in their capnoid and fatty acid chain compositions were identified. The SL called “sulfobacin B” was isolated, characterized, and was significantly increased in mice fed with high-fat diets. To reveal bacterial producers of SLs, metagenome analysis was acquired and only two bacterial genera, i.e., *Alistipes* and *Odoribacter*, were revealed to be responsible for their production. This knowledge enables explaining a part of the molecular complexity introduced by microbes to the mammalian gastrointestinal tract and can be used as chemotaxonomic evidence in gut microbiota.

## Introduction

Lipids are essential constituents of a complex organism involved in different biological functions^1, 2^. They contain fatty acids, phospholipids or sphingolipids as major classes of molecules. Sphingolipids include a variety of compounds, consisting of sphingomyelins, glycosphingolipids and ceramides, varying in the sphingoid backbone and fatty acid composition. Sulfonolipids (SLs) are an unusual class of sphingolipids with a sulfonic acid group in the sphingoid base and are structurally related to the class of ceramides^3^. Instead of sphingosine, capnine is the main precursor metabolite generated from cysteic acid and fatty acyl-CoA^4^. It has been reported that SLs, i.e., sulfobacin A, sulfobacin B and flavocristamide A, are produced by N-acylation of capnine with different iso-fatty acids^5^. SLs were reported in diatoms and particularly in some bacterial species including *Capnocytophaga*, *Cytophaga*, *Flexibacter* and *Sporocytophaga* strains^4, 6–11^. While investigating the components of cell surface, capnine and their N-acetylated SLs were found in *Capnocytophaga spp.* ^6^. Moreover, a related O-acetylated sulfonolipid subclass was described in halophilic bacteria namely S*alinabacter ruber* and *Salisaeta longa* ^12, 13^. In addition, a rosette inducing factor 1, as an N-acetylated SL compound is produced by a prey bacterium *Algoriphagus machipongonensis* ^14–17^.

In general, most of the bacteria containing SLs are from Bacteroidetes phylum, which is also an important component of intestinal ecosystem^18, 19^. Although sphingolipids are seldom detected in gut microbiota, some were found in genera of *Bacteroides, Prevotella* and *Porphyromonas* ^20–24^. We have reported previously that bacterial community and its metabolome is associated with body weight changes in two different mouse strains on a high-fat diet (HFD)^19^. Also, HFD was not only shown to elevate structurally related ceramides in liver as a proximal organ to gastrointestinal tract but also being a key trigger for altering gut bacterial communities^18, 25–27^. Furthermore, a shift towards Firmicutes is reported in HFD studies with a decrease in Bacteroidetes phylum^28^. It has been found that the potential sphingolipid producing genera such as *Bacteroides* or *Prevotella* are strongly associated with the lean phenotype in HFD studies^28, 29^.

In the present study, we have performed a detailed examination of bacterial SLs in murine cecum, accompanied by their screening, identification and characterization by combining liquid chromatography, mass spectrometry and nuclear magnetic resonance spectroscopy. We have also studied the profiles of SLs in a HFD mouse study (lard fat or safflower oil) and revealed that two bacterial genera including *Alistipes* and *Odoribacter* were responsible for their production. Accordingly, the analyses of cecal metagenomes permitted taxonomic elaboration of gene candidates in the sphingolipid biosynthetic pathway. Thus, by analysing the pure cultures of the proposed bacterial strains, we have confirmed the presence of SLs in those bacteria, while they were absent in germfree mice leading to the conclusion that bacteria in cecum are crucial for SLs generation *in-vivo*.

## Materials and Methods

### Animals

#### High-fat diet mouse study

Male C57BL/6NTac (C57BL/6N, Taconic Denmark) were bred under specific-pathogen-free conditions and housed under standard vivarium conditions (12:12 light dark cycle) in German Mouse Clinic. Mouse husbandry was conducted under a continuously controlled hygiene standard according to the Federation of European Laboratory Animal Science Associations. All animals received human care due to criteria outlined in the NAS “Guide for the Care and Use of Laboratory Animals”. Animal experiments were approved by the Upper-Bavarian district government (Regierung von Oberbayern, Gz.55.2-1-54-2532-70-07, Gz. 55.2-1-54-2532-4-11). In dietary challenge three different diets were applied, including a normal chow (NC; LFD, Diet#1310, Altromin, Germany; 17.0KJ/g, n = 7), experimental diets containing safflower oil (linoleic acid, SAFF, n = 9) or lard fat (oleic acid, LARD, n = 10) (HFD, Ssniff, Germany). High-fat diet study started at an age of 14 weeks with 3 weeks of intervention. Further details on diet composition are given elsewhere^30^. Body weight measurement was done at the beginning and the end of 3 weeks diet challenge. Then, mice were euthanized with isoflurane (Sigma Aldrich, St Louis, MO, USA), the luminal content of cecum of each mouse was collected, snap-frozen in liquid nitrogen, and stored at −80 °C.

#### Germfree, mono-colonized and specific-pathogen-free mice

Animal use was approved by the Upper-Bavarian district government (Regierung von Oberbayern, approval no. 55.2-1-54-2531-99-13). All animals were bred and housed in mouse facilities at the Technical University Munich (School of Life Sciences, Weihenstephan). C57BL/6N were kept under specific-pathogen-free conditions (SPF; n = 6) (12 h light/dark cycles at 24–26 °C) until 15 weeks of age. All mice were fed a normal chow (autoclaved Ssniff, Soest, Germany, R/M-H for SPF or M-Z V1124-300 for germfree animals) *ad libitum* and were sacrificed by carbon dioxide. Mice (n = 15) were made germfree (GF) by hysterectomy (Institute for Laboratory Animal Science, Hannover, Germany). Sterility was checked by cultivation of feces in LB or Wilkins-Chalgren Anaerobe Broth (WCAB) from Oxoid Limited (Thermo Fisher Scientific, Waltham, MA USA) every 10–14 days and at sampling, and microscopic observation of Gram-stained fecal smears was performed. A mold-trap was used to indicate presence of mold. Mono-colonization of GF mice with an *Alistipes* strain (CC-5826-WT-bac; DSM 27924) was done. Therefore, 100 µl of an overnight culture in WCAB medium was orally administered (approximately 10^8^ CFU/mouse) to 8 weeks old GF mice (n = 8). Colonization status in mice was checked every week by cultivation of collected feces pellets on WCA plates. Mice were sacrificed after 4 weeks of colonization. The values of the bacterial density of the cecal content were determined with the following averaged values of 1.16 × 10^12^ CFU/g of content. All experiments were performed in accordance within relevant guidelines and regulations.

### Bacterial strains

All strains (active cultures and cryocultures) were purchased from Leibniz Institute DSMZ – German Collection of Microorganisms and Cell cultures (Braunschweig, Germany). We derived following strains: *Parabacteroides distasonis* DSM 20701 (PDI), *Bacteroides thetaiotaomicron* DSM 2079 (BTH), *Bacteroides caccae* DSM 19024 (BCA), *Bacteroides uniformis* DSM 6597 (BUN), *Bacteroides vulgatus* DSM 1447 (BVU), *Parabacteroides goldsteinii* DSM 19448 (PGO), *Alistipes finegoldii* DSM 17242 (AFD), *Porphyromonas gingivalis* DSM 20709 (PGI), *Odoribacter splanchnicus* DSM 20712 (OSP), *Alistipes indistinctus* DSM 22520 (AIN), *Alistipes onderdonkii* DSM 19147 (AON), *Alistipes putredinis* DSM 17216 (APU), *Alistipes shahii* DSM 19121 (ASH), *Alistipes timonensis* DSM 25383 (ATI), *Alistipes obesi* DSM 25724 (AOB) and *Alistipes ihumii* DSM 26107 (AIH). Strains were grown under anaerobic conditions using Hungate method^31^ on a WCAB for at least three days at 37 °C. Two aerobic soil species were also purchased from DSMZ: *Chryseobacterium gleum* DSM 16776 (CGL) and *Flavobacterium johnsoniae* DSM 2064 (FJO). FJO was grown on Medium 67 (CY-Agar, DSMZ, Braunschweig, Germany). CGL was grown on LB Medium. Optical density was measured after three days of incubation. Incubation was performed in liquid media in Hungate tubes. Media contained 0.5 g/L L-cysteine (Sigma Aldrich, St Louis, MO, USA) to prevent oxygen and maintain reductive environment for bacterial growth.

### Chemicals

Methanol, acetonitrile and ammonium acetate were purchased from Sigma Aldrich (LC-MS CHROMASOLV®, FLUKA, Sigma Aldrich, St Louis, MO, USA). Glacial acetic acid (min. 99.95%) was purchased from Biosolve (ULC/MS, Valkenswaard, Netherlands).

### Sample preparation of cecal content of mice

Preparation of cecal samples was performed on dry ice to avoid bacterial metabolism (including cutting for weighing). Around 10 to 20 mg of frozen cecal content (wet weight) was transferred into sterile tubes containing ceramic beads to allow cell disruption (NucleoSpin^®^ Bead Tubes, Macherey-Nagel, Dueren, Germany). One mL of pre-chilled methanol (−20 °C) was added and homogenised for 5 min with 30 Hertz using TissueLyser (Qiagen, Retsch, Germany). Then, the samples were centrifuged (10 min, 20 800 g, 4 °C) and the supernatants were transferred into sterile microcentrifuge tubes, placed on ice. Four-fold sample dilution in methanol and volumes of 50 µL were used for FT-ICR-MS infusion and UHPLC-MS/MS analysis, respectively. In addition, a pooled sample was generated from all samples (n = 26) and the storage was at −80 °C in tightly closed tubes.

### Sample preparation of bacterial pellets and media

After 3 days of growth, bacterial media and pellets were separated by centrifugation (10 min, 2655 g, 4 °C). Pellet or 1 mL of bacterial medium was reconstituted in 1 mL of pre-chilled methanol (−20 °C), vortexed, and bacterial cells were disrupted by ultrasonic extraction for 15 min in ice-cold water as described elsewhere^32^. After centrifugation (10 min, 20 800 g, 4 °C), methanolic supernatants were used for mass spectrometric analysis. Short or long time storage was done at −20 °C or −80 °C. Transferring the samples to the vials was done on ice and the cooling temperature for all sample managers was set at +4 °C.

#### FT-ICR mass spectrometry of cecal samples

Ultrahigh-resolution mass spectra were acquired using a 12 Tesla solariX Fourier transform ion cyclotron resonance mass spectrometer (FT-ICR-MS; Bruker Daltonics GmbH, Bremen, Germany) coupled to an APOLLO II electrospray ionisation source (ESI). Diluted methanol extracts were injected into electrospray source using direct infusion and a microliter pump at a flow rate of 120 μL/h with a nebulizer gas pressure of 1.0 bar and a drying gas flow rate of 4 L/min. A source heater temperature of 200 °C was maintained to ensure rapid desolvation in the ionised droplets. The spectra were acquired with a time domain of 4 megawords and 500 scans were accumulated for each mass spectrum in a mass range from 140–1000 Dalton. All mass spectra were internally calibrated using fatty acids reference mass list. Data processing was conducted using Bruker Compass Data Analysis Version 4.2 (Bruker Daltonics GmbH, Bremen, Germany) and the chemical formulas assignment by an in-house made software (NetCalc)^19^.

#### UHPLC-TOF-MS/MS analysis of cecal samples

UHPLC-MS analysis was performed using a maXis instrument (time of flight MS (TOF)), (Bruker Daltonics, Bremen, Germany) in combination with an UHPLC system (Acquity, Waters, Eschborn, Germany) equipped with a photodiode array detector and an ACQUITY BEH C8 column (1.7 μm, 2.1 × 150 mm, Acquity, Waters, Eschborn, Germany). A solvent mixture of water/acetonitrile (A: 5 millimolar ammonium acetate/0.1% acetic acid in water; B: acetonitrile) was used. The gradient was as followed: starting conditions were 10% B, kept for 1 min, afterwards a first linear gradient was performed until 22% B for 2 min, then a second gradient was done for 4 min until 27% B, followed by 13 min of third gradient until 95% B. 95% B was kept for 2.5 min and then followed 0.1 min of going back to 10% B, which was hold 2.4 min. A pre-run time of 2 min was also set before every injection. Column oven temperature, injection volume at partial loop condition and flow rate were 60 °C, 5 μL, and 0.350 mL/min, respectively. Cooling temperature of the sample manager was set at + 4 °C. Mass spectra were acquired using MaXis TOF-MS in deprotonated mode ((−) TOF-MS). Samples were introduced into the ESI source at a nitrogen flow rate of 8 L/min (200 °C) with a nebulizer gas pressure of 2.0 bar and capillary voltage of 4000 V and acquisition rate of 5 Hz. Data processing was done using Compass DataAnalysis 4.2 and QuantAnalysis Version 2.2 (Bruker Daltonics GmbH, Bremen, Germany). Data dependent MS/MS experiments were performed in automated MS/MS mode of maXis instrument. Ions with intensity ≥1000 were subjected to MS/MS and were limited to 5 precursor ions in every MS scan. Furthermore, targeted MS/MS experiments were performed in Multiple Reaction Monitoring mode of maXis instrument for all SLs. Three different MS/MS fragmentation methods were established based on the elution profiles of the SLs. Information about retention time (rt) and parent-fragment ions (peak areas) were used as semi-quantitative data for statistical analysis (Table S2). Peak areas were picked with QuantAnalysis2.2. Peak areas were normalised to wet cecal content weight (mg) and autoscaled prior to multivariate statistical analysis.

### Isolation and characterization of SL2 and SL3 from OSP

OSP was grown anaerobically in a WCAB medium for 3 days at 37 °C and reached an OD650 of 1.9. Bacteria were separated from media by centrifugation and extracted by methanol (1 mL) as described above. Methanolic extracts from OSP were subjected to isolation and purification studies.

#### UHPLC-Iontrap mass spectrometry of bacterial samples

To achieve base-line separation, the UHPLC method for SLs detection was adapted based on the method described elsewhere^14^. For UHPLC separation C8 BEH column (1.7 µm, 2.1 × 150mm) was applied using a gradient of A:5 millimolar ammonium acetate/0.1% acetic acid in water and B:acetonitrile. We started with 65% B with an initial time of 3 min, and then it was increased to 100% B within 13 min and 99% B was held for 1 min, with reconditioning for 65% B of 2 min and a pre-runtime of 3 min. Flow rate was 0.250 mL/min and column temperature was 40 °C. Injection volume was 5 µL for OSP pellet. Cooling temperature of sample manager was +4 °C. Extracted pellet and collected fractions were measured with this method. Detection of SLs was performed by amaZon ETD iontrap mass spectrometer (Bruker Daltonics GmbH, Bremen, Germany) in negative ESI mode. Samples were introduced into ESI source at a nitrogen flow rate of 8 L/min (250 °C) with a nebulizer gas pressure of 2.0 bar, capillary voltage of 4500 V and acquisition rate of 52 Hz.

### Other methods

Details on HPLC-based separation and fractionation, as well as nuclear magnetic resonance spectroscopy, statistical analysis and pipeline of metagenomics are given in Supplementary Information.

## Results

### Screening and identification of SLs using FT-ICR-MS and UHPLC-MS/MS

**Figure 1:**
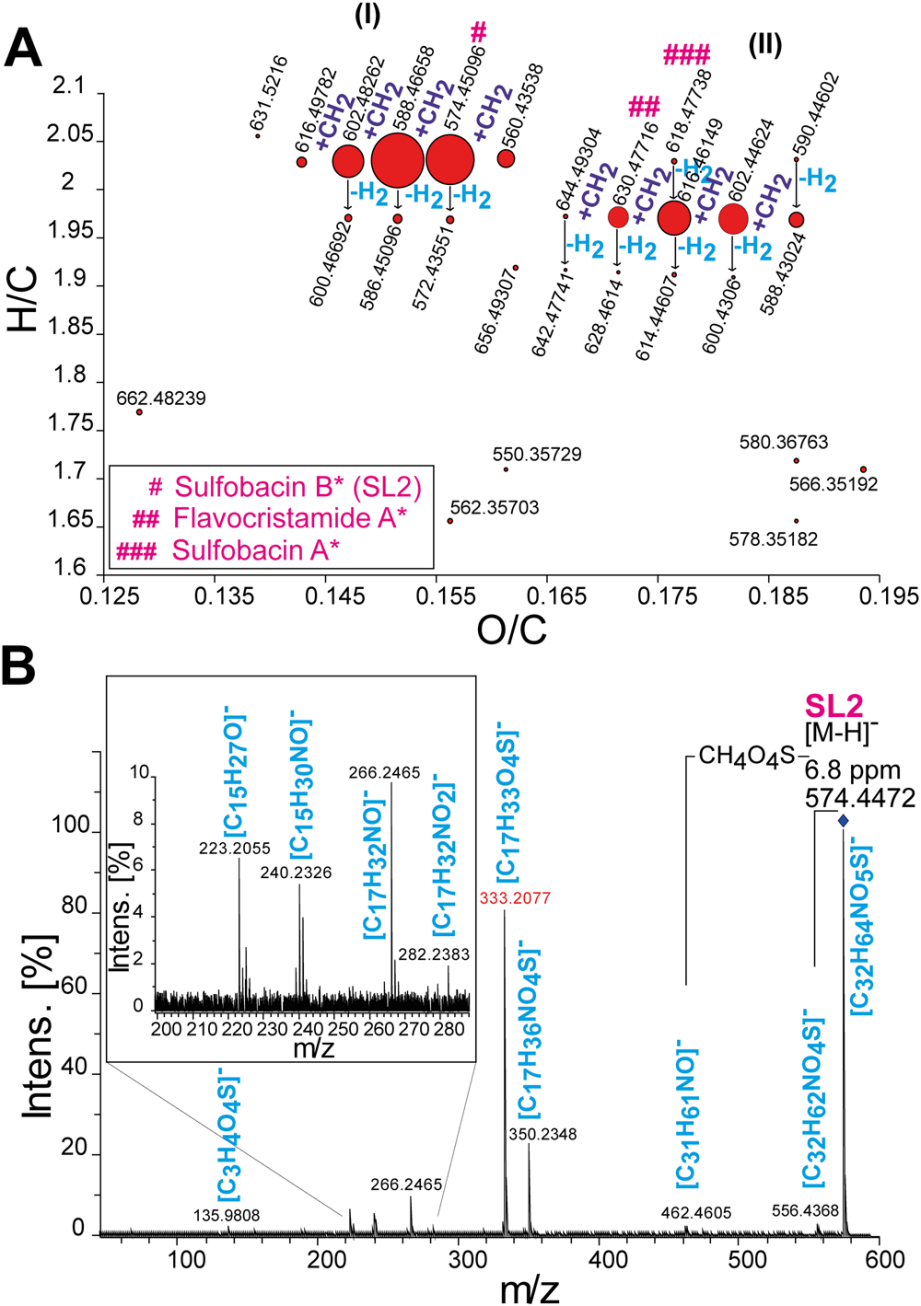
**Screening of the putative sulfonolipids (SLs) in gastrointestinal tract (cecum) of mice.** (**A**) Van Krevelen diagram of the SLs classes (I and II) as detected by ultrahigh-resolution FT-ICR-MS (insert stars reflect the putative annotation of the mass signals derived from ChemSpider database). (**B**) MS/MS spectrum of the SL2 and its corresponding fragment ions with their molecular formulas (blue color). Characteristic fragment ion of SL2 is highlighted in red.

In order to reveal the possible assignment and identification of SLs in cecal samples of C57BL/6N mice, we performed ultrahigh-resolution mass spectrometry FT-ICR-MS based experiments. Due to its high resolution (R ≥ 400000 at m/z = 400 and mass accuracy (up to an error ≤ 100 ppb), FT-ICR-MS provides highly valuable information about molecular compositions of most complex mixtures^33^. To screen possible SLs in gastrointestinal tract (GIT) of mice, extracts of cecal luminal content of 17 weeks old C57BL/6N mice (pooled sample of n = 7 mice) were analysed using FT-ICR-MS in deprotonated mode. As shown previously for sulfobacin A, B and flavocristamide A^5^, we agreed that the chemical formulas of the SLs share the following elemental composition (i.e., carbon C: 30 ≤ C ≤ 40, number of oxygen (O is 5 or 6), sulfur (S = 1) and nitrogen (N = 1)) in a nominal mass higher than 500 Da. We found 27 mass signals (m/z’s) that fulfil these criteria. To narrow down to the potential SLs, we plot all extracted chemical formulas in a van Krevelen diagram (Fig. 1A, red dots, size of dots represent relative intensity based on highest peak), where x- and y-axis relate to the O/C and H/C ratios, respectively (Fig. 1A). As shown in Fig. 1A (I) and (II), two classes of chemical formulas were observed. The horizontal and the vertical distributions differ due to the consecutive addition of methylene CH_2_ moieties (increasing values of x-axis) and hydrogens H_2_ moieties (decreasing values of y-axis) in each class (Fig. 1A, (I) and (II)). The difference between both classes is the number of oxygens, where class (I) contains 5 oxygen atoms and class (II) 6 oxygen atoms. Furthermore, the chemical formulas with H/C ratio ≤ 1.8 were excluded from further analysis (Fig. 1A, bottom), resulted in 21 different chemical formulas, representing possible SLs. As a representative of the class (I), we took the mass signal at m/z = 588.4665 with the corresponding chemical formula (C_33_H_66_O_5_N_1_S_1_, Fig. S1A(a)). To confirm the proposed chemical formula of 588.4665, isotopic fine structure (IFS) elucidation was investigated (Fig. S1A). Thus, the following isotopic chemical formulas [e.g., ^13^CC_32_H_66_O_5_N_1_S_1_ (Fig. S1A(b)), ^13^C_2_C_31_H_66_O_5_N_1_S_1_ and ^34^SC_33_H_66_O_5_N_1_ (Fig. S1A(c)), ^34^S^13^CC_32_H_66_O_5_N_1_ and ^18^O^13^CC_32_H_66_O_5_N_1_S_1_ (Fig. S1A(d))] were extracted and confirmed the presence of S in their chemical compositions. To associate the presence of the SLs in cecal extracts of mice and eliminate random hits, we performed additional analysis from seven individual cecal extracts (derived from C57BL/6N mice) using FT-ICR-MS (Fig. S1B). As shown in Fig. S1B, we could detect several mass signals corresponding to the following SLs putatively assigned to the sulfobacin A, B and flavocristamide A. Amongst the detected SLs, Godchaux *et al*. identified the precursor metabolite capnine, which is likely involved in the biosynthesis of SLs and similar to dihydrosphingosine, except the hydroxyl group at C1 position is substituted by sulfonate moiety^6^. Accordingly, a capnine compound with the chemical formula (C_17_H_37_O_4_N_1_S_1_) was detected in cecal samples of six mice with low abundant values compared to the intensity values of sulfobacin B (Fig. S1B). Table S1 summarize experimental mass signals that were detected in at least 5 mice with their corresponding generic name (if already reported), experimental mass values, molecular formulas and monoisotopic masses. In total, eighteen of the twenty-one mass signals were detected and fulfil the established chemical criteria for SLs (Table S1). Aligned peak lists are given in SI for m1-m7 mice with mass signals and individual intensities of SL1-SL18 that are highlighted in grey. In addition, UHPLC-MS/MS analysis of the pooled sample of cecal content (n = 26 mice) resulted in a base peak chromatogram (BPC) characteristic of this sample (Pooled sample, shown in Fig. S1C). We were able to identify 9 different features which correspond to the mass signals of SL1-SL9 within a mass window of ±0.005 Dalton (Table S1 and Fig. S1C). Furthermore, MS/MS based experiments enable to identify structural composition of SL1-SL9. For instance, MS/MS of SL2 and its detailed fragmentation pathway as well as the common substructure of SLs are shown in Fig. 1B and Fig. S2. Extensive MS/MS elaboration showed the presence of eighteen SLs. We determined the unique MS/MS fragment for each SL which was used for peak area integration in the following experiments (Fig. S3; A-H). As shown in Fig. S3A, SL1 is dissociating into two characteristic fragment masses, i.e., 347.2232 and 333.2076 corresponding to the chemical formulas C_18_H_35_O_4_S^−^ and C_17_H_33_O_4_S^−^ as fragments of capnoid base, respectively (Fig. S3A). Thus, we distinguish between SL1:1 and SL1:2 based on the MS/MS experiments of the parent and the fragment ion mass signals (Table S2).

### Diet alters occurrence of sulfonolipids in cecal samples of C57BL/6N mice

**Figure 2:**
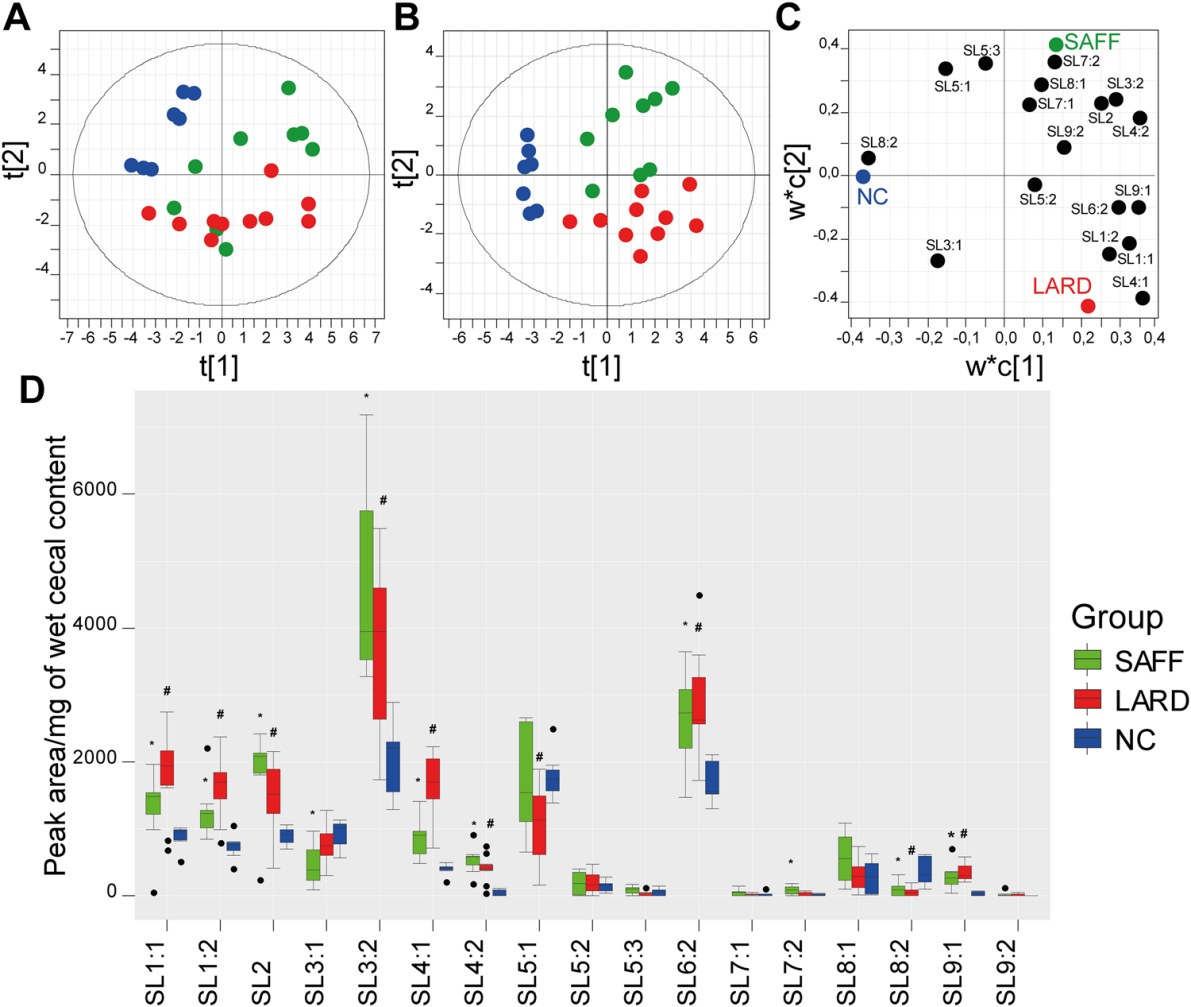
**High-fat diet alters SLs in cecum of C57BL/6N mice.** (**A**) PCA displaying SAFF (green dots; n = 9), LARD (red dots, n = 10) and NC (blue dots; n = 7) diet fed mice. Data is derived by integrating peak areas of all SLs, taking parent-fragment ion information as described in Table S2. (**B**) PLS-DA of SAFF, LARD and NC samples, with their respective loadings plot of SLs (black dots), as shown in (**C**). (**D**) Individual box plots of each SL and their distribution in each group (Welch t-test; P-value < 0.05; *SAFF vs. NC; #LARD vs. NC).

The main factor responsible for influencing gut microbiota is diet, especially high-fat diet, which leads to body weight increase and obesity, accompanied by a change in the composition of gut microbiota. We suggest that dietary changes altering gut microbiota would lead to a subsequent change in the relative abundance of SLs in the cecum of mice. To determine whether SL levels are diet dependent, we fed a C57BL/6N mouse strain with two experimental high-fat diets containing either safflower oil (linoleic acid as main fatty acid, SAFF) or lard fat (oleic acid as main fatty acid, LARD) as a fat source during 3 weeks of intervention at 14 weeks of age, comparing this to a normal chow (NC) diet. This resulted in a significantly higher body weight in the LARD and SAFF group, compared to the NC group (p-value < 0.05, body weights of all mice are listed in Table S3). Using UHPLC-MS/MS analysis of cecal samples, peak areas of all SLs were integrated and the characteristic of their parent-fragment ions are summarized in Table S2. Figure 2A shows normalized and autoscaled peak area data of eighteen SLs using principal component analysis (PCA; SAFF = green dots; LARD = red and NC = blue dots). The PCA was able to describe 62.7% of the total variance where the first component t[1] and the second component t[2] represent 39.2% and 23.5% of this variance, respectively. To reveal, which SLs are responsible for discriminating SAFF, LARD and NC samples, we have applied partial least squares to latent structures discriminant analysis (PLS-DA) with R^2^Y and Q^2^CUM values of 0.686 and 0.536, respectively (score plot is shown in Fig. 2B). In loading plot of PLS-DA, we could illustrate which SLs (black dots) are clustering according to each diet (Fig. 2C) and we have observed that almost all SLs are increased in high-fat fed mice. Thus, SL1:1, SL1:2, SL2, SL3:2, SL4:1, SL4:2, SL6:2 and SL9:1 were significantly increased in SAFF and LARD fed mice, while SL7:2 was only significantly increased in SAFF fed mice compared to NC fed mice (Fig. 2C). Accordingly, SL3:1, SL5:1 and SL8:2 were higher in NC fed mice. It needs to be mentioned that the pellets of high-fat diets did not show any presence of the identified SLs. Individual boxplots for each lipid and their corresponding significance are shown in Fig. 2D. Thus we can conclude that diets alter the SL patterns analysed in cecal contents of C57BL/6N mice.

### Metagenomic elaboration of bacterial candidates of SLs production

**Figure 3:**
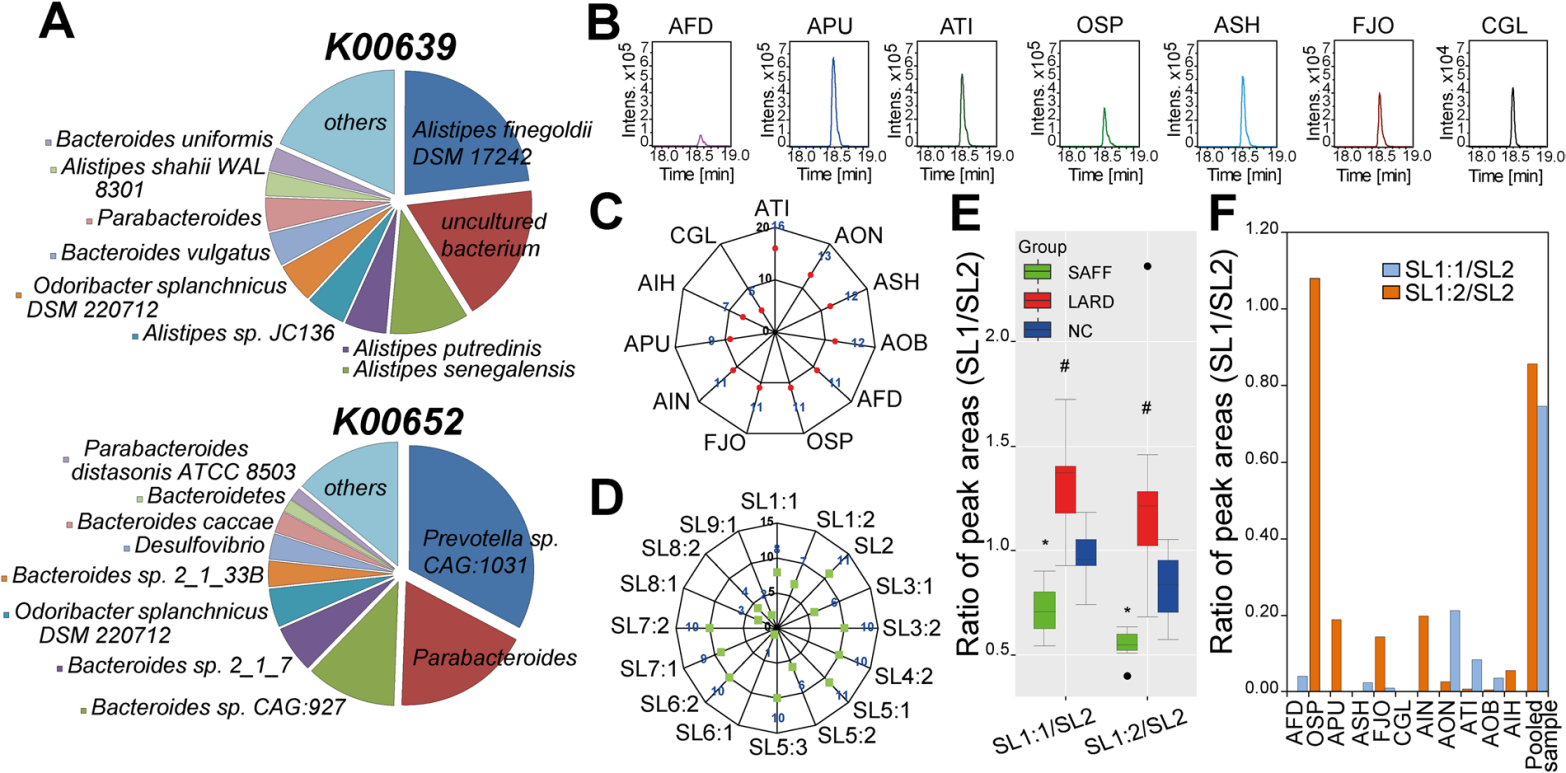
**K00639 is key gene to predict SLs producers by metagenomics.** (**A**) Taxonomic distribution of bacterial sphingolipid biosynthesis genes K00639 and K00652 for SAFF mice (n = 6), that were elaborated by metagenomics. (**B**) LC peak of SL2, found in AFD (pink), APU (blue), ATI (dark green), OSP (green), ASH (light blue), FJO (brown) and CGL (black), respectively. (**C**) Radar diagram of the detected SLs in bacteria (number of detected SLs for each strain is indicated in blue). (**D**) Radar diagram of each SL and number of bacteria, in which each lipid was identified. (**E**) Ratios of SL1:1/SL2 and SL1:2/SL2 that are significantly altered between SAFF (black), LARD (red) and NC (blue) fed mice (Welch t-test; P-value < 0.05; *SAFF vs. NC; #LARD vs. NC). (**F**) Ratio analysis of SL1:1/SL2 (orange) and SL1:2/SL2 (blue), as well as their distribution in bacterial cultures of *Alistipes* strains (AFD, APU, ATI, ASH, AIN, AON, AOB and AIH), OSP, FJO, CGL and a pooled sample of cecal content extract (Pooled sample; n = 26).

Several bacteria were found to produce SLs such as gliding bacteria *Cytophaga*, or other bacteria such as *Flexibacter*, *Sporocytophaga*, *Capnocytophaga, Salinibacter *and *Algoriphagus* ^6–8, 15, 34^. To investigate, which bacterial strains produce SLs in the GIT, we have performed a metagenome analysis and screened for bacterial genes potentially involved in SL biosynthesis in the cecum of C57BL/6N mice. De-novo synthesis of SLs is thought to be similar to ceramide synthesis, which is a part of sphingolipid biosynthesis^35^. The key gene in the sphingolipid biosynthesis is serine palmitoyltransferase (SPT; K00654, EC 2.3.1.50, Sphingolipid metabolism). It has been reported that SPT possesses several bacterial gene homologues with high E-values such as 5-aminolevulinate synthase (K00643, EC 2.3.1.29, Glycine, serine and threonine metabolism; Porphyrin and chlorophyll metabolism), 2-amino-3-ketobutyrate coenzyme A ligase (K00639, EC 2.3.1.29, Glycine, serine and threonine metabolism) or 8-amino-7-oxononanoate synthase (K00652, EC 2.3.1.47, Biotin metabolism)^35^. Metagenomics were performed for SAFF mice (n = 6) and revealed gene sequences of K00639 and K00652 that were present in the cecal gut microbiome (Fig. 3A). Gene sequences of SPT and K00643 were not detected by metagenome analysis. The bacterial candidates with the highest scores and abundances are shown in a pie diagram (Fig. 3A; K00639 and K00652). These were *Alistipes finegoldii* DSM 17242 (AFD), *Alistipes senegalensis*, *Alistipes putredinis*, *Alistipes* sp. JC136, *Odoribacter splanchnicus* DSM 220712 (OSP), *Bacteroides vulgatus*, *Parabacteroides*, *Alistipes shahii* WAL8301 and *Bacteroides uniformis* for K00639. For K00652, we found several potential bacteria, including *Prevotella* sp. CAG: 1031, *Parabacteroides*, *Bacteroides* sp. CAG:927, *Bacteroides* sp. 2_1_7, OSP, *Bacteroides* sp. 2_1_33B, *Desulfovibrio*, *Bacteroides caccae*, Bacteroidetes and *Parabacteroides distasonis* ATCC 8503. The following bacterial candidates were cultivated and analysed for SLs: AFD, *Alistipes putredinis* DSM 17216 (APU), *Alistipes* sp. JC136 (*Alistipes timonensis* DSM 25383; ATI), *Alistipes shahii* WAL8301 (*Alistipes shahii* DSM 19121; ASH), OSP, *Bacteroides vulgatus* DSM 1447 (BVU), *Parabacteroides distasonis* DSM 20701 (PDI), *Bacteroides uniformis* DSM 6597 (BUN) and *Bacteroides caccae* DSM 19024 (BCA). Moreover, as a positive control for SLs production, we have cultivated two aerobic soil species including *Flavobacterium johnsoniae* DSM 2064 (FJO) and *Chrysobacterium gleum* DSM 16776 (CGL) which were reported as producers of SLs^8, 36^. AFD, APU, ATI, OSP and ASH are able to synthesize SLs amongst the reported soil species FJO and CGL (Fig. 3B). All other cultivated bacteria, in which these lipids were not detected, are shown in Fig. S4A (LC Peak of SL2 is shown as a representative SL, measured in bacterial pellets of AFD, BVU, PDI, BUN and BCA). We conclude that taxonomical assignment for gene sequence of K00639 is the most promising gene candidate to predict SLs producers. We could observe that SLs were detected only in bacterial pellets but not in their supernatant, as shown for SL2 of AFD (Fig. S4B). Due to fact that AFD was tested positively for SLs, we obtained four further *Alistipes* strains, including *Alistipes indistinctus* DSM 22520 (AIN), *Alistipes onderdonkii* DSM 19147) (AON), *Alistipes obesi* DSM 25724 (AOB) and *Alistipes ihumii* DSM 26107 (AIH), to measure SLs and all of them were found to produce SLs (SL2 is shown in Fig. S4C). Other representatives of Bacteroidetes were also tested such as *Parabacteroides goldsteinii* DSM 19448 (PGO), *Bacteroides thetaiotaomicron* DSM 2079 (BTH), *Porphyromonas gingivalis* DSM 20709 (PGI), which were non-producers of SLs compared to AFD (EICs of SL2 as a representative are shown in Fig. S4D).

All 18 SLs were screened in bacterial cultures by UHPLC-MS/MS to obtain a deeper insight into all SLs produced in the *Alistipes* genera, one *Odoribacter* species and two soil species. From these 18 identified SLs in cecum, only 16 SLs were detected in bacteria and followed this order: ATI > AON > (ASH, AOB) > (AFD, OSP, FJO, AIN) > APU > AIH > CGL (Fig. 3C). SL4:1 and SL9:2 were not detected in our bacterial cultures, while SL2 and the capnine precursor metabolite were detected in all 11 analysed strains (Fig. 3D, Table S4). Furthermore, 10 strains were able to produce SL3:2, SL4:2, SL5:3, SL6:2 and SL7:2 (Fig. 3D). All identified SLs are summarised in Table S4. The data, concerning extracted ion chromatograms of SL1-SL9 as well as their MS/MS are shown in Fig. S5.

In addition, we have observed different behavior when comparing peak areas of SL1:1 and SL1:2 to SL2. In cecal samples, the average ratio of SL1:1/SL2 was 1.03, with significantly higher values in LARD mice, compared to SAFF and NC mice (Fig. 3E). Similarly, the ratio of SL1:2/SL2 has a mean of 0.91 and was significantly decreased in SAFF mice compared to LARD and NC mice (Fig. 3E). We have observed a ratio of SL1:1/SL2 between 0 and 0.3 for bacterial strains compared to a pooled sample of cecal extracts with a ratio of 0.85 (Fig. 3F). On the hand, the ratio of SL1:2/SL2 ranged between 0 and 1 for bacteria. Except OSP, none of other bacteria reached a ratio > 0.3 for this SL ratio comparison (Fig. 3F).

Furthermore, SL2 and SL3 were isolated from OSP and characterized by nuclear magnetic resonance (NMR) spectroscopy. SL2 was characterized as sulfobacin B with C17:0 capnoid unit and a C15:0 fatty acid chain^8^, whereas SL3 was characterized as a new SL with a C17:0 capnoid unit and a C17:0 fatty acid chain containing a keto-group at the 2′ position in the fatty acid unit (Fig. S6–9, and Tables S5–6). Both SLs contain iso-methyl-branched fatty acids (Fig. S6B).

### SLs are absent in germfree mice

**Figure 4:**
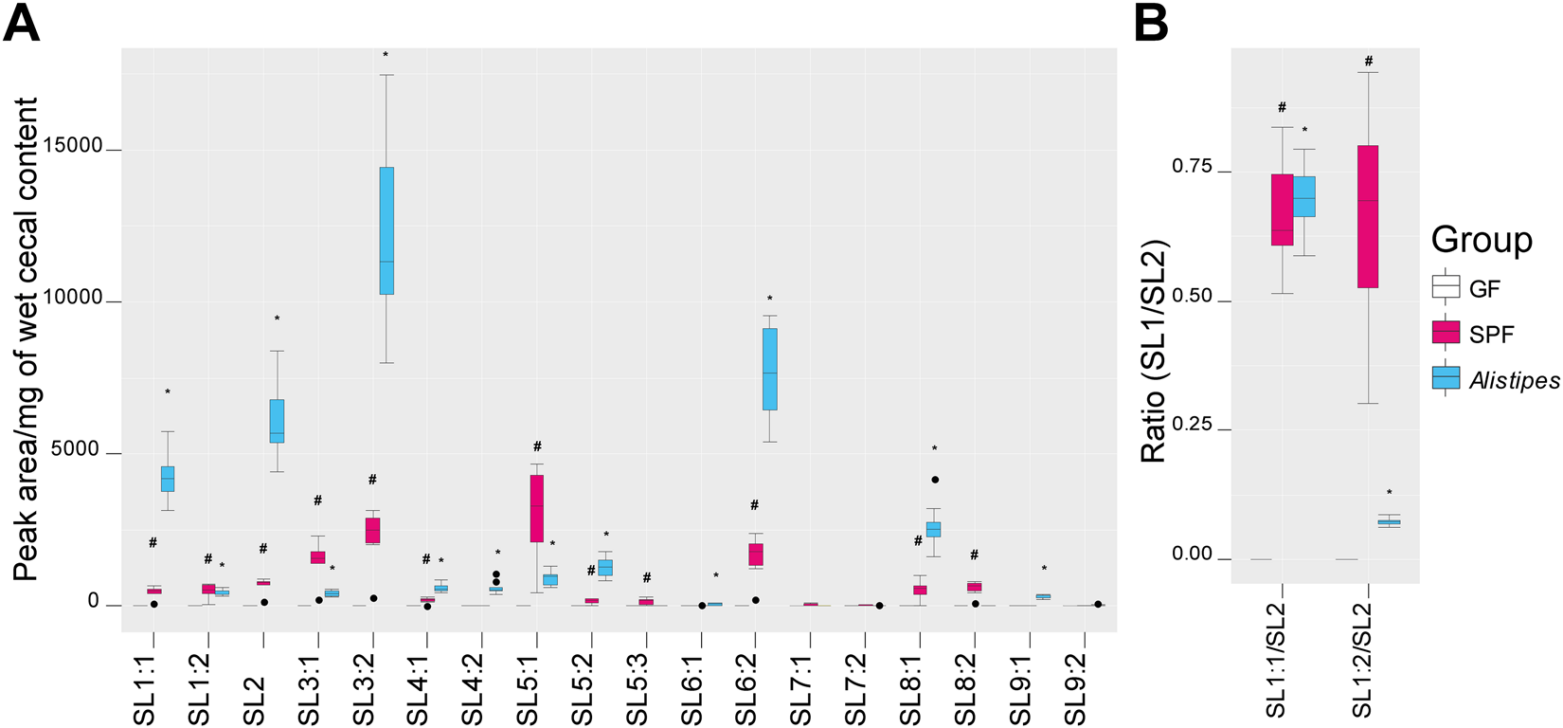
**Gut microbiota is essential to detect SLs in-vivo.** (**A**) SLs are absent in germfree (GF, white; n = 7) mice, comparing SPF mice (magenta; n = 6) and mono-colonized mice with *Alistipes* sp. strain (CC-5826-WT-bac) (turquoise; n = 8). (**B**) Ratio comparison of SL1:1/SL2 and SL1:2/SL2. (Welch t-test; P-value < 0.05; #GF vs. SPF; *GF vs. *Alistipes*).

To verify whether SLs are of bacterial origin and not a result of host metabolism, we obtained germfree and SPF mice to investigate this hypothesis. All identified SLs were exclusively produced in SPF mice (magenta) as compared to germfree mice (GF, white) (Fig. 4A; all data of peak areas are summarised in Table S7). Moreover the ratio of SL1:1/SL2 or SL1:2/SL2 was on average 0.65, and significantly increased in SPF mice compared to GF mice (Fig. 4B). To further confirm the production of SLs by certain bacteria, we performed a mono-colonization of GF mice with *Alistipes* sp. strain (CC-5826-WT-bac; DSM 27924, turquoise) and we have observed a significant appearance of SLs in the cecum of mono-colonized mice as compared to GF mice (Fig. 4A). Thus, the ratio of SL1:1/SL2 was comparable to those calculated in SPF mice, concluding that one species is enough to reach this ratio (Fig. 4B). In contrast, the ratio SL1:2/SL2 was much higher in SPF mice, indicating that complex gut microbiota in SPF mice have additional bacterial strains that could be liable to the production of SLs.

## Discussion

This study revealed that unusual sulfonolipids (SLs) are a part of cecal luminal content, collected from mice. Until now, only environmental bacteria were found to contain SLs^1, 6, 7, 9, 12, 14, 15, 34, 36, 37^. They were never described in mammals, and are considered as a rare class of sphingolipids with unknown biological functions. We have identified SLs bearing a sulfonic acid group based on their elemental formula compositions and LC-MS/MS experiments. Thus, we have narrowed down the potential candidates based on the previously reported molecular formulas of sulfobacin A, B and flavocristamide A supported by the isotopic fine structure analysis^5^. With a total set of eighteen SLs, we have investigated whether these lipids are changed in mice fed with either safflower oil or lard fat based high-fat diet (HFD). HFDs have not only a huge impact on body weight, obesity and coincident diseases but do also influence lipid metabolism such as glycerophospholipid, sphingolipid and glycerolipid levels^38–40^. In our study, almost all SLs were increased in HFD groups as was the case for sulfobacin B (SL2). This result could be argued that fatty acids delivered by food are used as a source to generate bacterial lipids. In addition, we have confirmed that SLs are a part of two important core bacterial genera of the mouse gut microbiome^41^. *Alistipes* and *Odoribacter*, members of the Bacteroidetes phylum, were both found to produce SLs, with and without mammalian host. We have detected SLs in pure cultures of *Odoribacter splanchnicus* DSM 220712 and eight *Alistipes* species. Interestingly, it has been reported that sphingolipids are exclusively produced in eukaryotes due to the lack of the key enzyme serine palmitoyltransferase^23, 42^. Accordingly, our metagenome data showed the presence of two bacterial gene homologues including 2-amino-3-ketobutyrate coenzyme A ligase (K00639) and 8-amino-7-oxononanoate synthase (K00652) but no genes of serine palmitoyltransferase^35^. However, recently several bacterial species have been reported to produce different sphingolipid molecules, such as ceramide phosphorylethanolamines, ceramide phosphoglycerols or galactosylceramides^24, 42, 43^. For instance, human symbiotic gram-negative bacteria, i.e., *Bacteroides, Parabacteroides, Porphyromonas* and *Prevotella*, contain sphingolipids, whereas *Alistipes* were stated to be non-producers^23, 24, 43^. We have revealed that some strains of these genera (*Bacteroides*, *Parabacteroides* and *Prevotella*) were also found to have serine palmitoyltransferase gene homologues might be potentially responsible for the production of other sphingolipid related molecules^24, 42, 43^. In addition, sphingolipids identified in *Bacteroides fragillis* execute different biological functions, such as induction of immune system or maintain bacterial survival and stress resistance in intestine of mammals^23, 44, 45^. As an example, glycosylated sphingolipids, such as alpha-galactosylceramide, bind to CD1d of dendritic cells and activate both mouse and human invariant natural killer T-cells^46, 47^.

Some roles of SLs were already investigated and showed bioactivity, while performing antagonistic action on Willebrand factor receptors, inducing cytotoxicity in cancer cells, exhibiting inhibitory functions of DNA polymerase, and demonstrating anti-inflammatory effects^8, 36, 48^. In a mouse model of lipopolysaccharide induced acute-inflammation, a suppression of tumor necrosis factor alpha was shown for sulfobacin B^36^. Furthermore, SLs isolated from *Algoriphagus* induce rosette-shaped multicellular colonies of choanoflagellates, showing potential signaling function in developmental processes^14–17^. Genera of *Alistipes* are mostly associated with mammalian habitats and were already reported to be altered in cancer, irritable bowel syndrome, animal based diet, HFD or vegetables consumption^18, 29, 49–52^. Recently, a study showed a direct connection between *Alistipes finegoldii* and improvement of DSS-induced colitis^53^. SLs of *Alistipes* and *Odoribacter* show great prospects for potentially biological readout, such as the maturation of intestine or immune system. However, there is still a big gap in knowledge and more investigation is needed to determine the biochemical and functional roles of these bacterial lipids from both genera.

In summary, we have presented here a novel class of sphingolipid compounds, namely sulfonolipids, which are identified for the first time in a mammalian gut system with profiles related to different high-fat diets. Two different genera *Alistipes* and *Odoribacter* were responsible for the production of SLs. This new finding allows a broader understanding of the chemical complexity of these bacterial lipids. The strategy of thorough SL analysis by mass spectrometry has given an insight into sulfur-bearing lipids of two different genera in the phylum of Bacteroidetes. This class of lipids is a very promising bacterial metabolite marker and could be used as chemotaxonomic information in following prospective studies of gut microbiota performed by metabolomics or lipidomics approaches.

## Electronic supplementary material

Supplementary Information

Supplementary Dataset 1
