# Supplementary material for: Sulfonolipids as novel metabolite markers of Alistipes and Odoribacter affected by high-fat diets
Source: Sci Rep. 2017 Sep 8;7:11047. doi: 10.1038/s41598-017-10369-z (PMC5591296; doi:10.1038/s41598-017-10369-z)
Supplement: Supplementary file 1 — Supplementary Information [file 41598_2017_10369_MOESM1_ESM.pdf]

Supporting Information

Sulfonolipids as novel metabolite markers of *Alistipes* and *Odoribacter* affected by high-fat diets

Alesia Walker[1*], Barbara Pfitzner[2], Mourad Harir[1,6], Monika Schaubeck[3], Jelena Calasan[3], Silke S. Heinzmann[1], Dmitrij Turaev[4], Thomas Rattei[4], David Endesfelder[5], Wolfgang zu Castell[5], Dirk Haller[3,7], Michael Schmid[2], Anton Hartmann[2], Philippe Schmitt-Kopplin[1,6,7*]

[1]Research Unit Analytical BioGeoChemistry, Helmholtz Zentrum München, German Research Center for Environmental Health, Neuherberg, Germany

[2]Research Unit Microbe-Plant Interactions, Research Group Molecular Microbial Ecology, Helmholtz Zentrum München, German Research Center for Environmental Health, Neuherberg, Germany

[3]Chair of Nutrition and Immunology, Technische Universität München, Freising-Weihenstephan, Germany

[4]Division of Computational System Biology, Department of Microbiology and Ecosystem Science, University of Vienna, Vienna, Austria

[5]Scientific Computing Research Unit, Helmholtz Zentrum München, German Research Center for Environmental Health, Neuherberg, Germany

[6]Chair of Analytical Food Chemistry, Technische Universität München, Freising-Weihenstephan, Germany

[7]ZIEL – Institute for Food & Health, Technische Universität München, Freising-Weihenstephan, Germany

Corresponding authors:
Alesia Walker & Philippe Schmitt-Kopplin
Helmholtz Zentrum München
German Research Center for Environmental Health
Department of Environmental Sciences (DES)
Analytical BioGeoChemistry (BGC)
Ingolstaedter Landstrasse 1
85764 Neuherberg
Germany
Tel.: (+49) 089 3187 4656
E-Mail: alesia.walker@helmholtz-muenchen.de
E-Mail: schmitt-kopplin@helmholtz-muenchen.de

Running title: Unusual sulfonolipids as a part of gut microbiota

Key words: metabolomics, sulfonolipids, sulfobacin B, capnines, UHPLC-MS, FT-ICR-MS, gut microbiota, metagenomics, *Alistipes*, *Odoribacter*

Supplementary materials and methods

High pressure liquid chromatography-based separation and fractionation

Fractionation experiments were performed on an Agilent 1290 Infinity LC system using an Acquity Xbridge™ column (5µm, 4.6 x 250 mm, Waters, Germany). A gradient of water/acetonitrile (A, 5 millimolar ammonium acetate/0.1% acetic acid in water; B, acetonitrile) was used for the fractionation experiments. The gradient used was 65% (B) for 8.40 min, and was increased to 99% (B) within 30 min and then held for 2.40 min. Recondition was done for 5 min with a pre-runtime of 8 min to 65% (B). The flow rate, the column temperature and the injection volume were 1 mL/min, 40°C and 100µL, respectively. Sample manager was cooled to +4°C. The fractions were collected every minute using 215 Liquid Handler (Gilson, France), resulting in a total of 25 individual fractions. Detection of SLs in bacterial extract and fractions was performed by amaZon ETD ion trap mass spectrometer (Bruker Daltonics GmbH, Bremen, Germany) coupled to UHPLC. The extracts as well as each fraction were analyzed using UHPLC-Iontrap-MS in negative ionization mode using similar method as mentioned in Materials and Methods (main text).

NMR Spectroscopy.

Fractions 10 and 11 representative of SL2 and SL3 (Supplementary Figure 4A) were evaporated (SpeedVac Concentrator, Savant SPD121P, Thermo Scientific) and reconstituted in 200 µL DMSO-d6 with addition of trimethylsilyl-tetradeuteropropionic acid (TSP), as a reference standard. One dimensional proton (^{1}H)- NMR spectra were acquired on a Bruker 800 MHz spectrometer (Bruker Biospin, Rheinstetten, Germany) operating at 800.35 MHz with a quadruple inverse cryoprobe at 300 K. A standard 1D pulse sequence [recycle delay (RD)- 90°-t1-90°-tm-90°-acquire free induction decay (FID)] was acquired, with water suppression irradiation during RD of 2 s, mixing time (tm) set on 100 ms, and a 90° pulse set to 10.13 µs, collecting 800 scans into 64,000 data points with a spectral width of 12 ppm. In addition, a 2D total correlation spectroscopy (TOCSY) analysis was performed, using a 1H-1H phase-sensitive sensitivity-improved 2D pulse sequence with water suppression by gradient tailored excitation (3-9-19) and DIPSI-2. 19,228 × 1,024 data points were collected using 32 scans per increment, an acquisition time of 1 s, and 16 dummy scans. Spectral widths were set to 12 ppm in the F2 and F1 dimensions. Processing of spectrum was performed using TopSpin 3.2 (Bruker BioSpin).

FIDs were multiplied by an exponential decaying function corresponding to a line broadening of 0.3 Hz (F1) and 2.5 Hz (F2) before Fourier transformation, manual phasing, baseline correction and calibration to TSP (δ 0.00) was also performed in TopSpin. Chemical shifts, multiplicity and J-coupling constants were compared to Kamiyama et al.[1] and predicted spectra using ACD/NMR prediction software (ACD/Labs, Toronto, ON, Canada).

Statistical analysis

SIMCA-P version 9.0 (Umetrics, Umea, Sweden) was used for the principal component analysis (PCA) and the partial least squares (PLS) plots to latent structures discriminant analysis. Other illustrations and univariate statistics were done using Microsoft Office (Excel), R (https://www.rstudio.com/) and SigmaPlot 12.0 (Systat Software, Inc, San Jose, CA).

Metagenomics

Metagenomic studies were made only from C57BL/6NTac mouse group fed a safflower enriched high fat diet. In total 10 metagenomes were prepared: from 6 mice cecal samples were used (6 biological replicates), 4 of them could be prepared as duplicates (4 technical replicates). Genomic DNA was extracted from cecal luminal content (30mg) using an extraction kit NucleoSpin96 for Soil according to protocol. DNA was quantified using Quant-iT™ PicoGreen® dsDNA Kit. Sequencing was done by applying a whole-genome sequencing approach on the GS-FLX+ Titanium™ sequencing platform from Roche (Roche Diagnostics GmbH, Mannheim, Germany). DNA libraries were prepared for each metagenome on 1 µg of sample DNA following manufacturer's instructions. After nebulisation DNA fragments were processed by end repair, adapter ligation and size selection. Products were purified and quantified. Quality assessment of libraries on an Agilent Bioanalyzer High Sensitivity DNA Chip (Agilent Technologies, Santa Clara, USA) determined fragment lengths of sequence libraries of around 1400 bp, which were taken for further sequencing. By titration mainly a 6-12 DNA-copies per bead ratio was determined. After emulsion PCR and subsequent bead recovery enrichments of 790 000 DNA-beads were pooled per sample and loaded onto each quarter of a PicoTiter-Plate. Sequencing of long fragments was applied by selecting a 200 cycles sequencing run. Metagenome sequence data are available on Sequence Read Archive (SRA) under BioProject ID PRJNA299870. For quality control, prinseq-lite was used: Three bases were trimmed from the 5' end, bases with a quality score <20 in a

window of 3 bases were trimmed from the 3' end, and sequences with a mean quality <20 were discarded[2]. Minimum length of all sequences was restricted to 150 bp, maximum length to 500 bp. Contaminating mouse DNA sequences were detected by a sequence similarity search using BLASTN (NCBI BLAST 2.2.26+ max. e-value 0.1, DUST filter off) of all sequences against mouse reference genome on NCBI (build GRCm38.p1)[3,4]. An alignment length of ≥80% of the query sequence and e-values $\leq 10^{-4}$ were used as cutoff criteria; sequences matching these criteria were considered presumptive mouse sequences and removed. To determine taxonomic origin of sequences and associated gene functions, a sequence similarity search was performed using BLASTX (NCBI BLAST 2.2.26 with the -w 15 parameter set, allowing for frameshifts in alignments, max. e-value 10) against the NCBI non-redundant (NR) database (downloaded 07/19/2013)[3]. Output was imported into MEGAN 5 (version 5.7.1), using parameters min. bitscore 50, max. e-value $\leq 10^{-2}$. Functional gene annotation was performed in MEGAN using KEGG classification of reads[5]. Based on RefSeq-IDs mapping to KEGG orthology (KO) groups, each read was mapped to a gene with KO identification. Taxonomical assignment of genes involved in sphingolipid metabolism was determined by MEGAN [6].

Supplementary figures

Figure S1

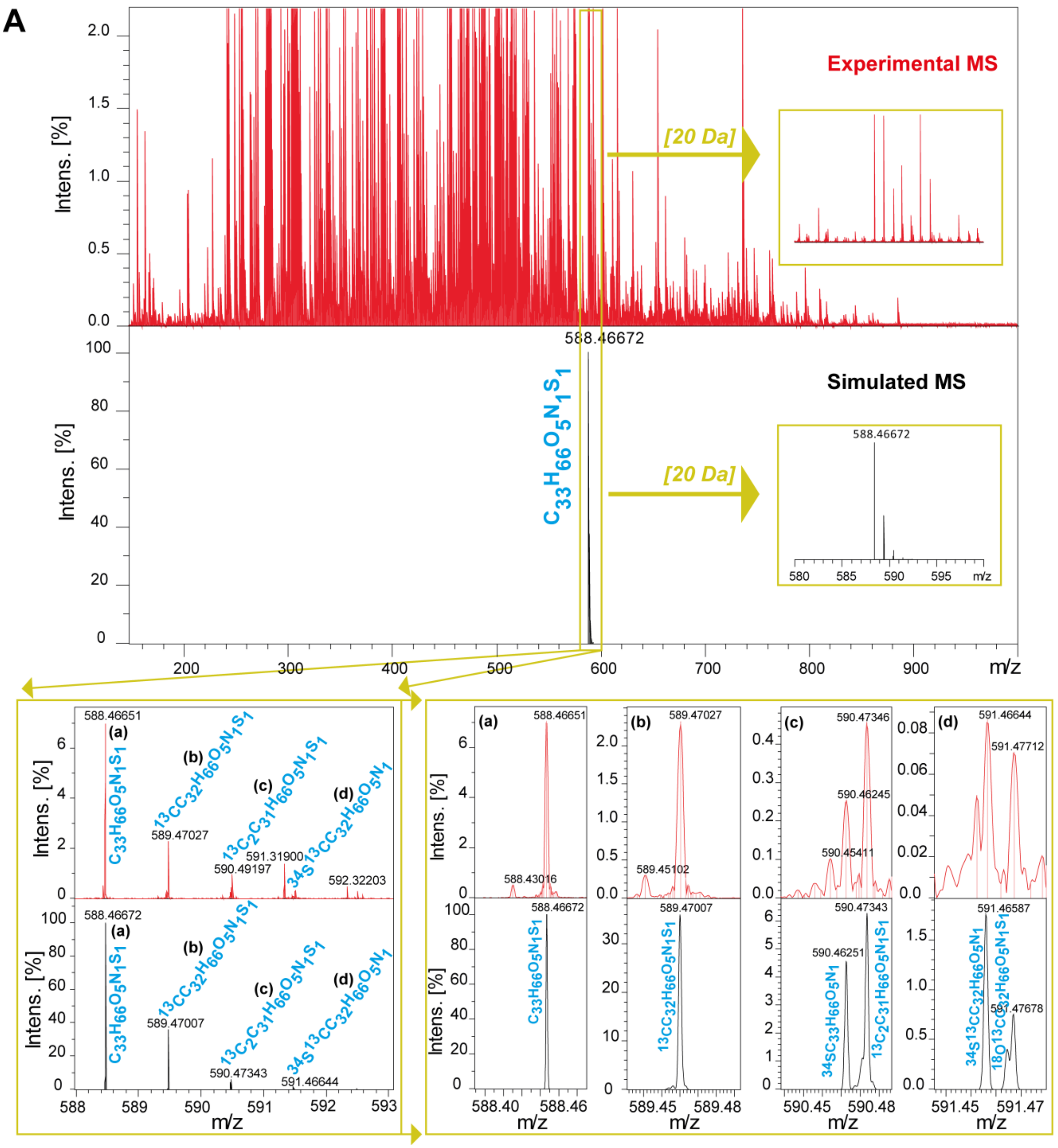

Figure S1 (continued).

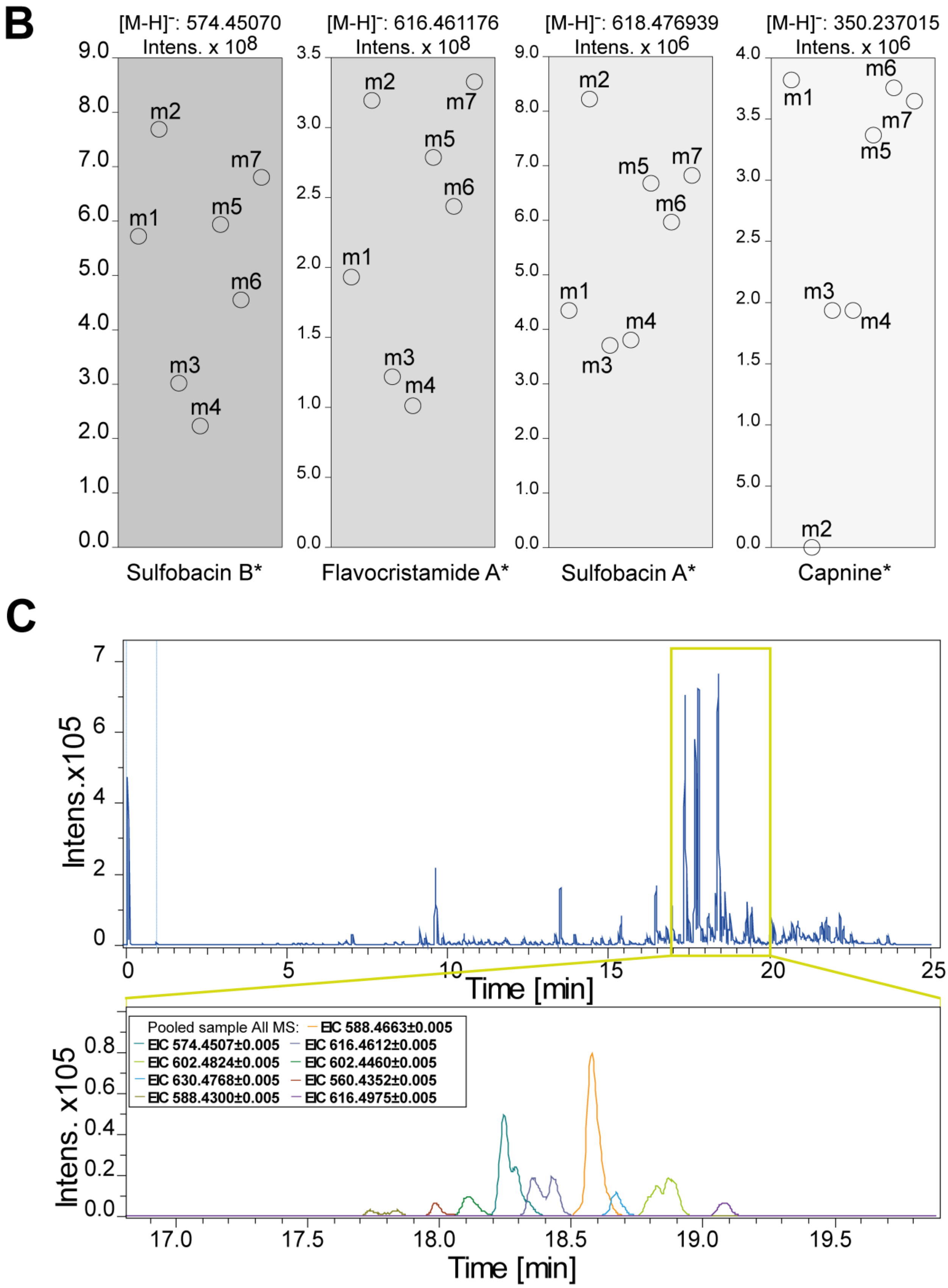

Figure S1.

(A) Screening of SLs using FT-ICR-MS, showing a full MS spectrum (120-1000 Dalton) with a zoomed insert figure of 20 Dalton in the range of putative SLs (580-600). Isotopic fine structure analysis of a putative SL compound with m/z = 588.44651 and assigned molecular formula $C_{33}H_{66}O_5N_1S_1$ (a). Insert molecular formulas i.e., $^{13}CC_{32}H_{66}O_5N_1S_1$ (b), $^{13}C_2C_{31}H_{66}O_5N_1S_1$ and $^{34}SC_{33}H_{66}O_5N_1$ (c), $^{34}S^{13}CC_{32}H_{66}O_5N_1$ and $^{18}O^{13}CC_{32}H_{66}O_5N_1S_1$ (d) show its corresponding chemical isotopologues, respectively. Validation was done by comparing experimental mass signals versus simulated mass signals**.** (B) shows a [ESI(-)]FT-ICR-MS data of three mass signals corresponding to possible SLs (sulfobacin B, flavocristamide A, sulfobacin A; SL1, SL3 and SL13 in Table S1), measured in cecum of seven individual mice (m1-m7), together with their putative precursor compound capnine ($C_{17}H_{37}NO_4S$; Chemspider ID: 169799) putatively assigned based on molecular formula search in ChemSpider. (C) Chromatogram of pooled sample. Insert figure shows extracted ion chromatograms of 9 peak signals corresponding to SL1-SL9 compounds within an extraction error window of ±0.005 Dalton (see also Table S1).

Figure S2

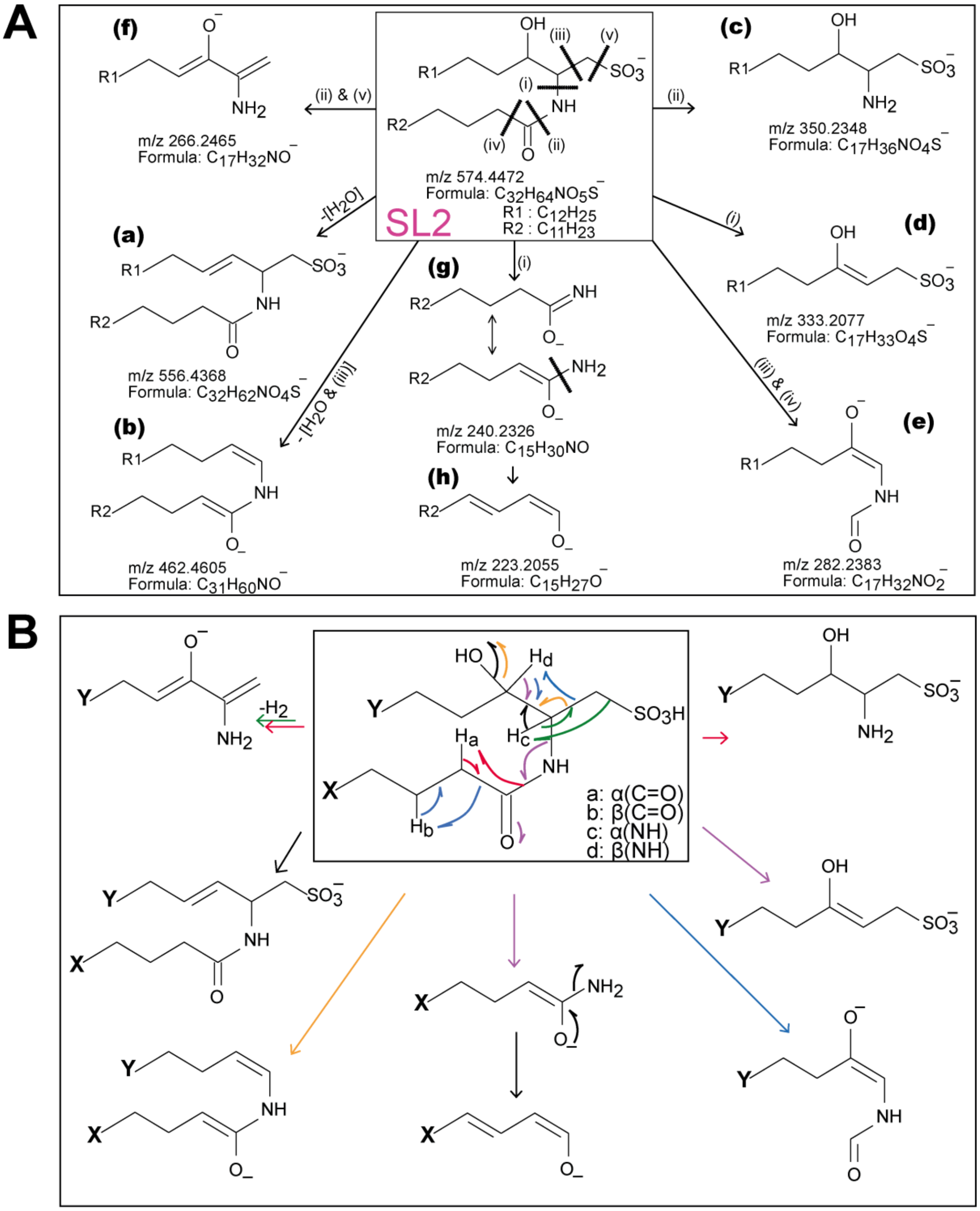

Figure S2.

(A) and (B) show ion fragmentation pathways and mechanism of formation of main ion fragments of SL2 compound, respectively. Fragmentation pathways of SL2 compound is described as following. MS/MS fragments of the sulfonolipid SL2 shows existence of fragment ion (a) m/z=556.4368-[$C_{32}H_{62}NO_4S^-$] which could be elucidated by a dehydration of SL2 m/z=574.4472-[$C_{32}H_{64}NO_5S^-$]

(Figure 2A). Accordingly, fragment ions (f) m/z=266.2485-[$C_{17}H_{32}NO^-$] and (e) m/z=282.2363-[$C_{17}H_{32}NO_2^-$] could be generated in two steps by loss of HSO_3H and CH_3SO_3H moieties as well as transfer of protons γ and β to amine and ketone groups respectively (Figure S2A). Furthermore, transfer of proton α to ketone group led to formation of the fragment ion (c) m/z=350.2348-[$C_{17}H_{36}NO_4S^-$] (Figure S2A). In addition, fragment ion (b) m/z = 462.4605-[$C_{31}H_{60}NO^-$] could be obtained by loss of $HOCH_2SO_3H$ moiety (Figure S2A). Transfer of proton β to amine group followed by rearrangement to amide group leads to fragment ions (d) m/z=333.2077-[$C_{17}H_{33}O_4S^-$] and (g) m/z=240.2326 [$C_{15}H_{30}NO^-$]. Further dissociation of fragment ion (g) m/z=240.2326-[$C_{15}H_{30}NO^-$] by loss of ammonia moiety leads to fragment ion m/z=223.2056-[$C_{15}H_{27}O^-$]. Most of these pathways employ following bond-ruptures namely carbon-oxygen (C-O), carbon-nitrogen (C-N), carbon-sulfur (C-S) and nitrogen-carbonyl (N-C=O). Furthermore, most of these bond-ruptures engage transfer of proton α-β to carbonyl (C=O) and α-β-γ to amine (NH) groups followed by rearrangement based reactions. Details on mechanisms of the fragmentation pathways are illustrated in Figure S2B.

Figure S3

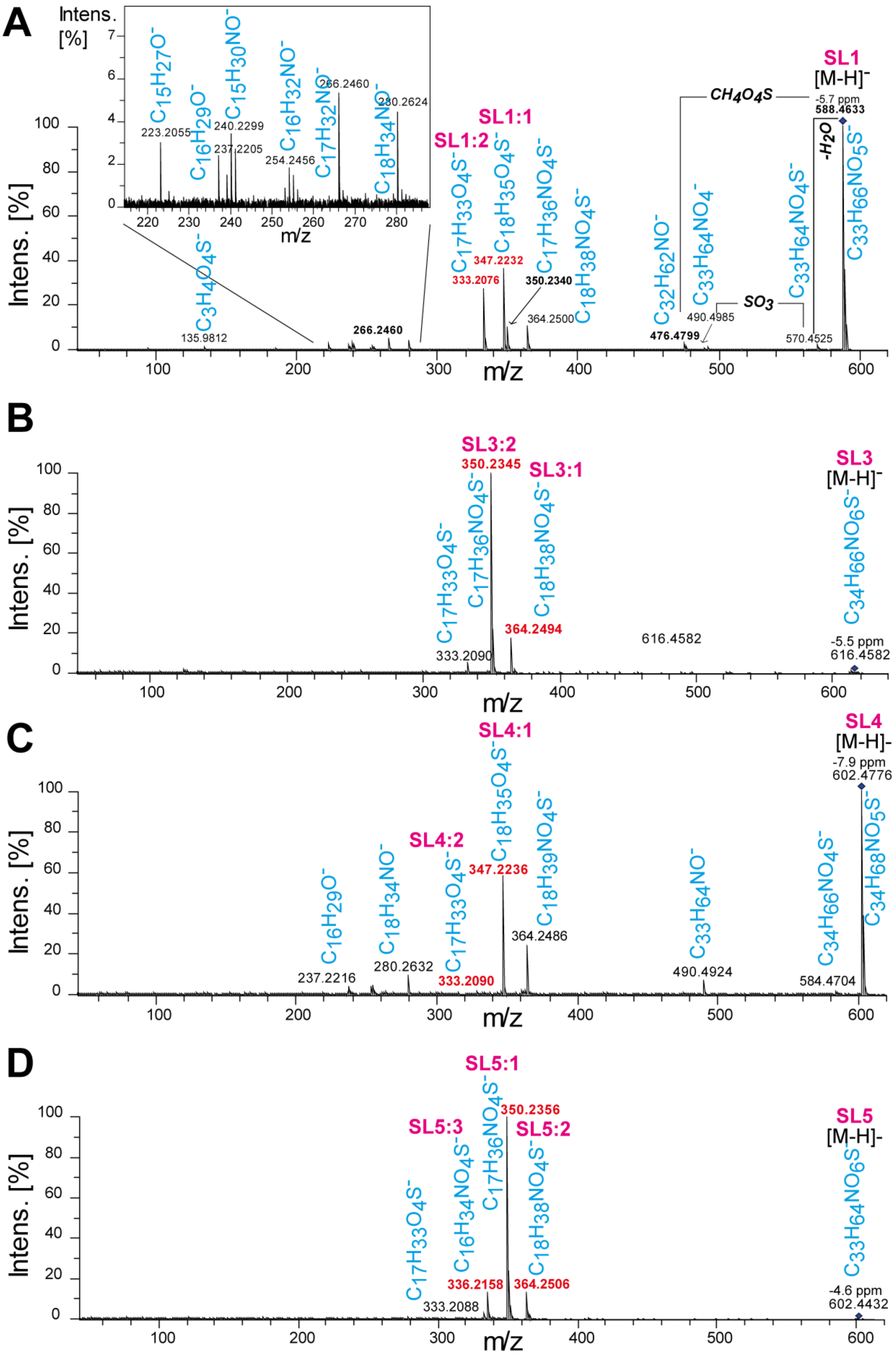
A
B
C
D
SL1
[M-H]-
SL1:1
SL1:2
SL3
SL3:1
SL3:2
SL4
SL4:1
SL4:2
SL5
SL5:1
SL5:2
SL5:3
Intens. [%]
m/z
CH4O4S
SO3
-H2O
588.4633
-5.7 ppm
616.4582
-5.5 ppm
602.4776
-7.9 ppm
602.4432
-4.6 ppm
347.2232
333.2076
350.2340
364.2500
266.2460
476.4799
490.4985
570.4525
135.9812
350.2345
364.2494
333.2090
347.2236
364.2486
280.2632
237.2216
490.4924
584.4704
350.2356
336.2158
364.2506
333.2088
223.2055
240.2299
237.2205
254.2456
280.2624

Figure S3 (continued)

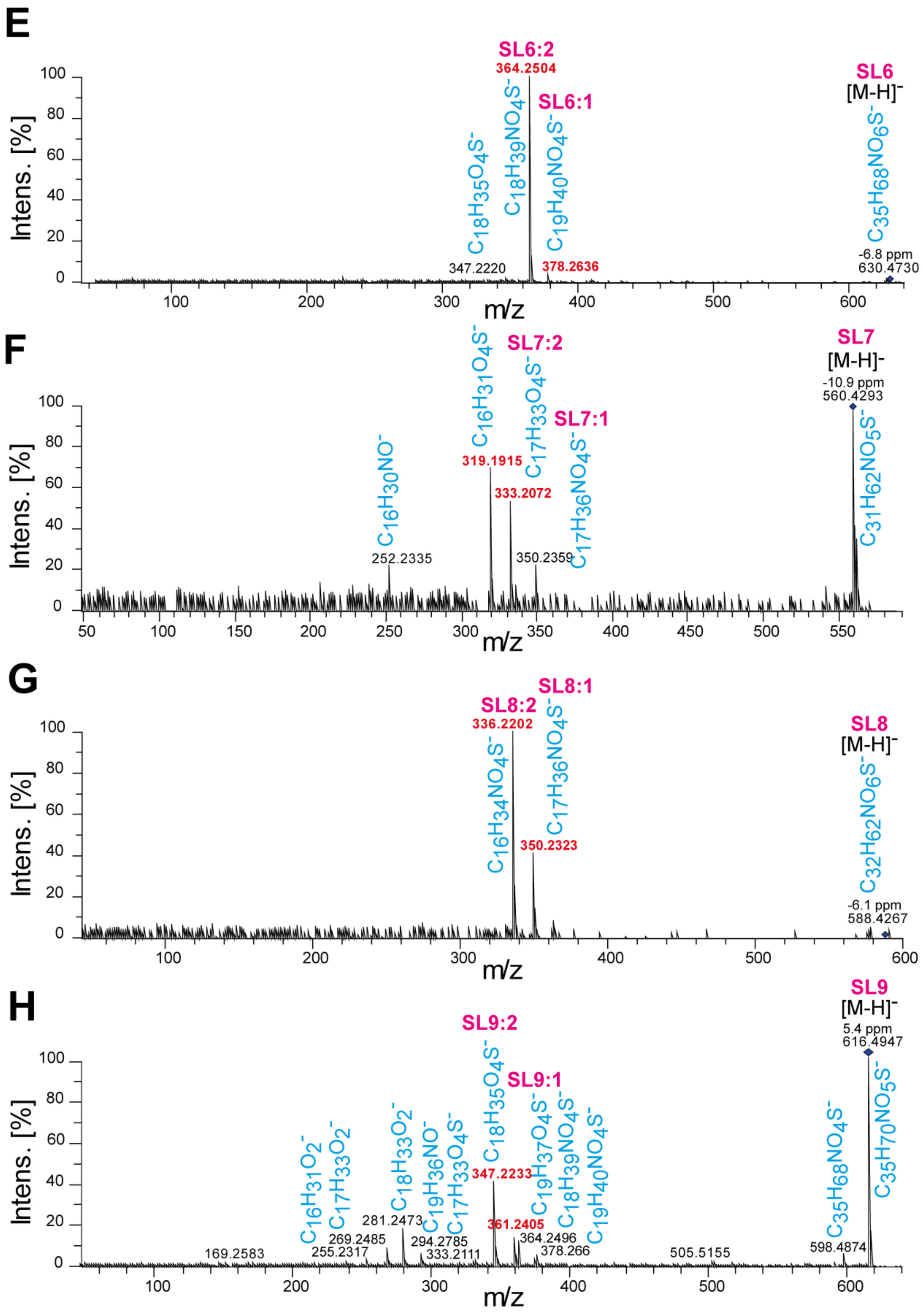

Figure S3.

MS/MS spectra of all identified SLs compounds including SL1 and SL3-SL9. Masses of ion fragments used for peak area integration are highlighted in red color with their corresponding SL number in pink color**.**

Figure S4.

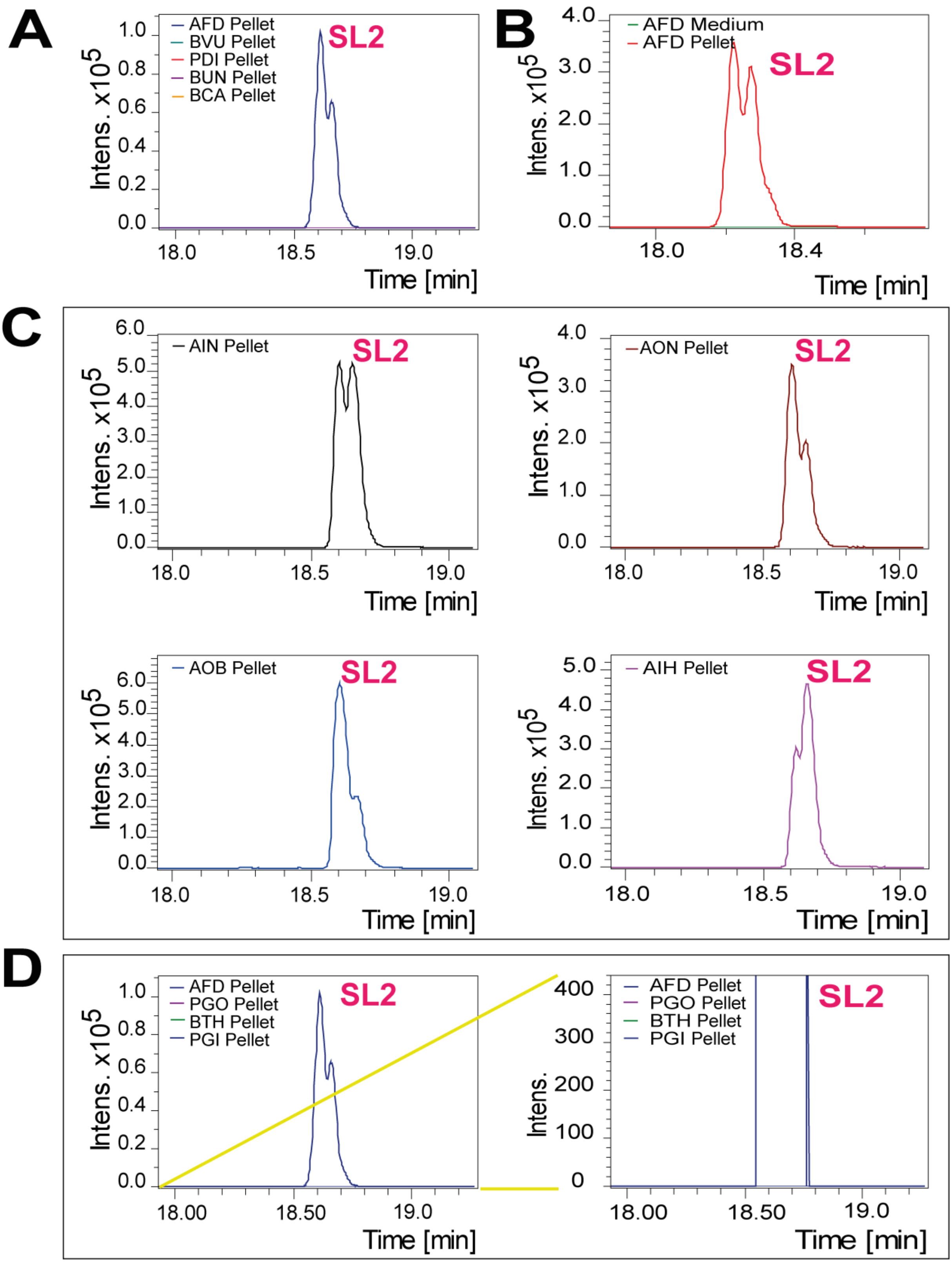

A
AFD Pellet
BVU Pellet
PDI Pellet
BUN Pellet
BCA Pellet
SL2
Intens. x10^5
Time [min]
B
AFD Medium
AFD Pellet
SL2
Intens. x10^5
Time [min]
C
AIN Pellet
SL2
AON Pellet
SL2
AOB Pellet
SL2
AIH Pellet
SL2
Intens. x10^5
Time [min]
D
AFD Pellet
PGO Pellet
BTH Pellet
PGI Pellet
SL2
Intens. x10^5
Intens.
Time [min]

Figure S4.

Extracted ion chromatograms of SL2: (A) SL2 peak in AFD, compared with non-producing bacteria such as BVU, PDI, BUN, BCA. (B) Peak of SL2 detected in AFD medium or pellet. (C) Peak of SL2 of four other Alistipes strains (AIN, AON, AOB and AIH). (D) Peak of SL2 in AFD, compared with other measured non-producing bacteria such as PGO, BTH and PGI with detailed inspection in intensity area of 400.

Figure S5.

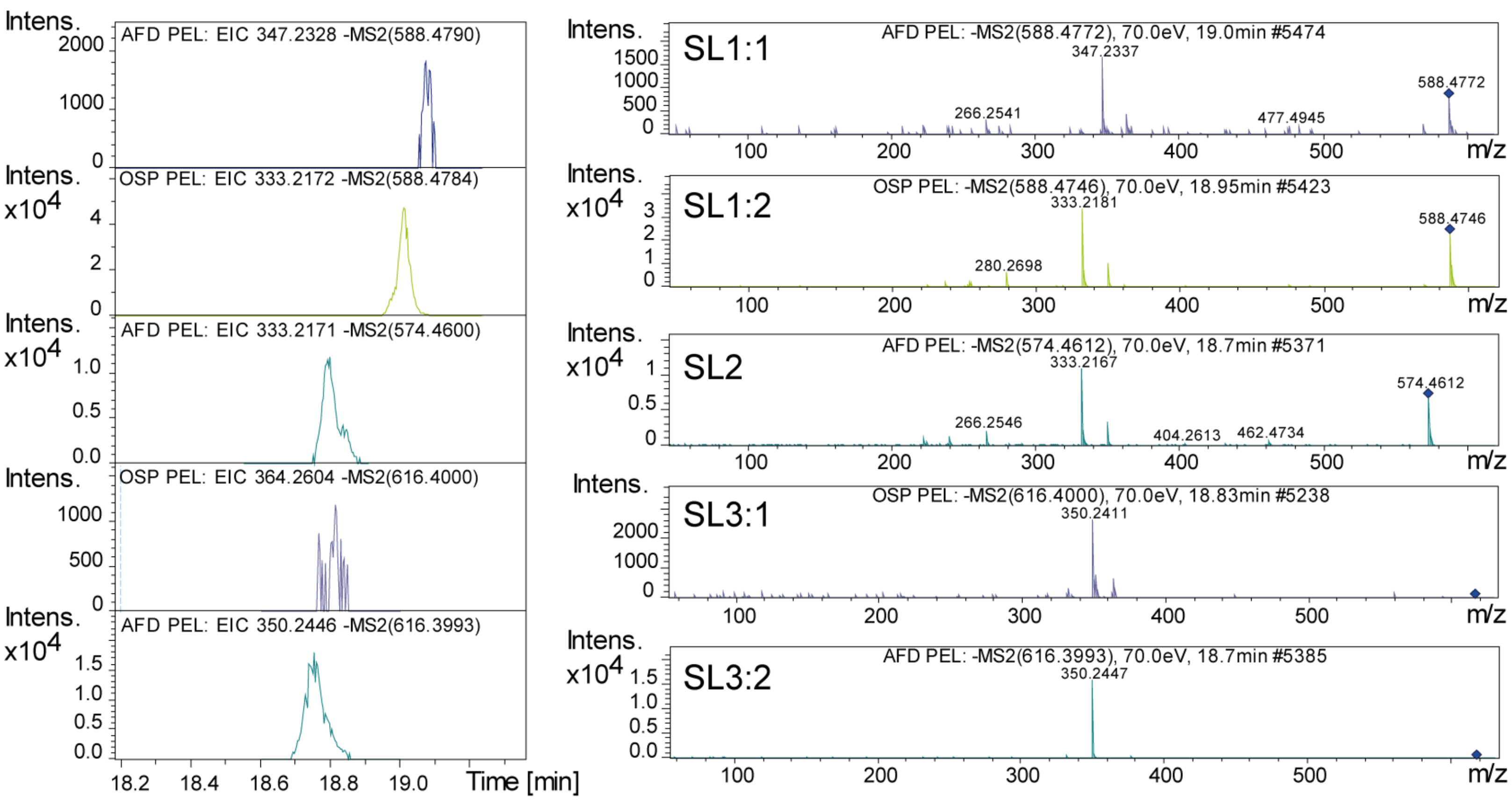
Intens.
AFD PEL: EIC 347.2328 -MS2(588.4790)
OSP PEL: EIC 333.2172 -MS2(588.4784)
AFD PEL: EIC 333.2171 -MS2(574.4600)
OSP PEL: EIC 364.2604 -MS2(616.4000)
AFD PEL: EIC 350.2446 -MS2(616.3993)
x10^4
18.2
18.4
18.6
18.8
19.0
Time [min]
SL1:1
AFD PEL: -MS2(588.4772), 70.0eV, 19.0min #5474
347.2337
588.4772
266.2541
477.4945
SL1:2
OSP PEL: -MS2(588.4746), 70.0eV, 18.95min #5423
333.2181
588.4746
280.2698
SL2
AFD PEL: -MS2(574.4612), 70.0eV, 18.7min #5371
333.2167
574.4612
266.2546
404.2613
462.4734
SL3:1
OSP PEL: -MS2(616.4000), 70.0eV, 18.83min #5238
350.2411
SL3:2
AFD PEL: -MS2(616.3993), 70.0eV, 18.7min #5385
350.2447
m/z

Figure S5 (continued)

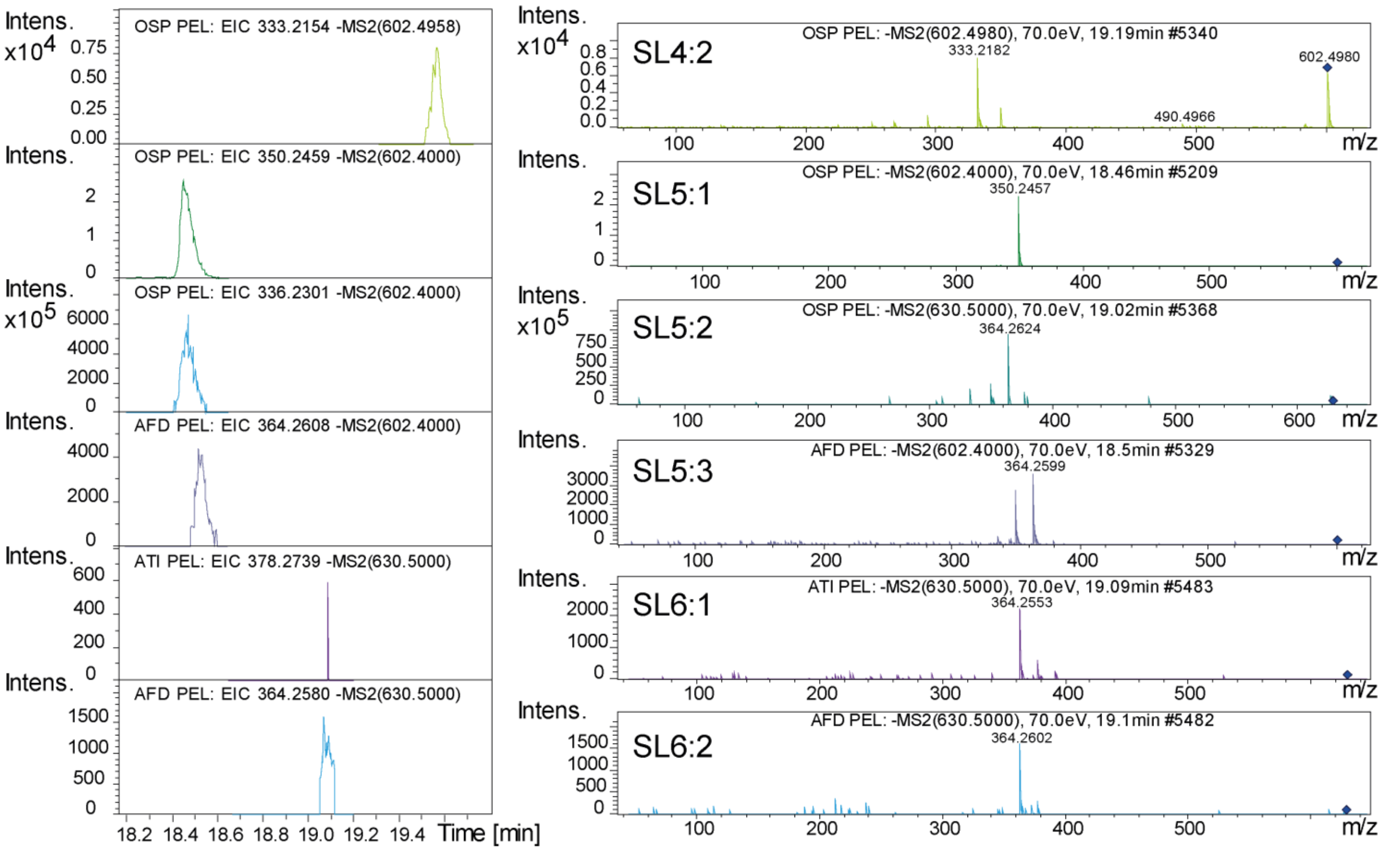

Figure S5 (continued).

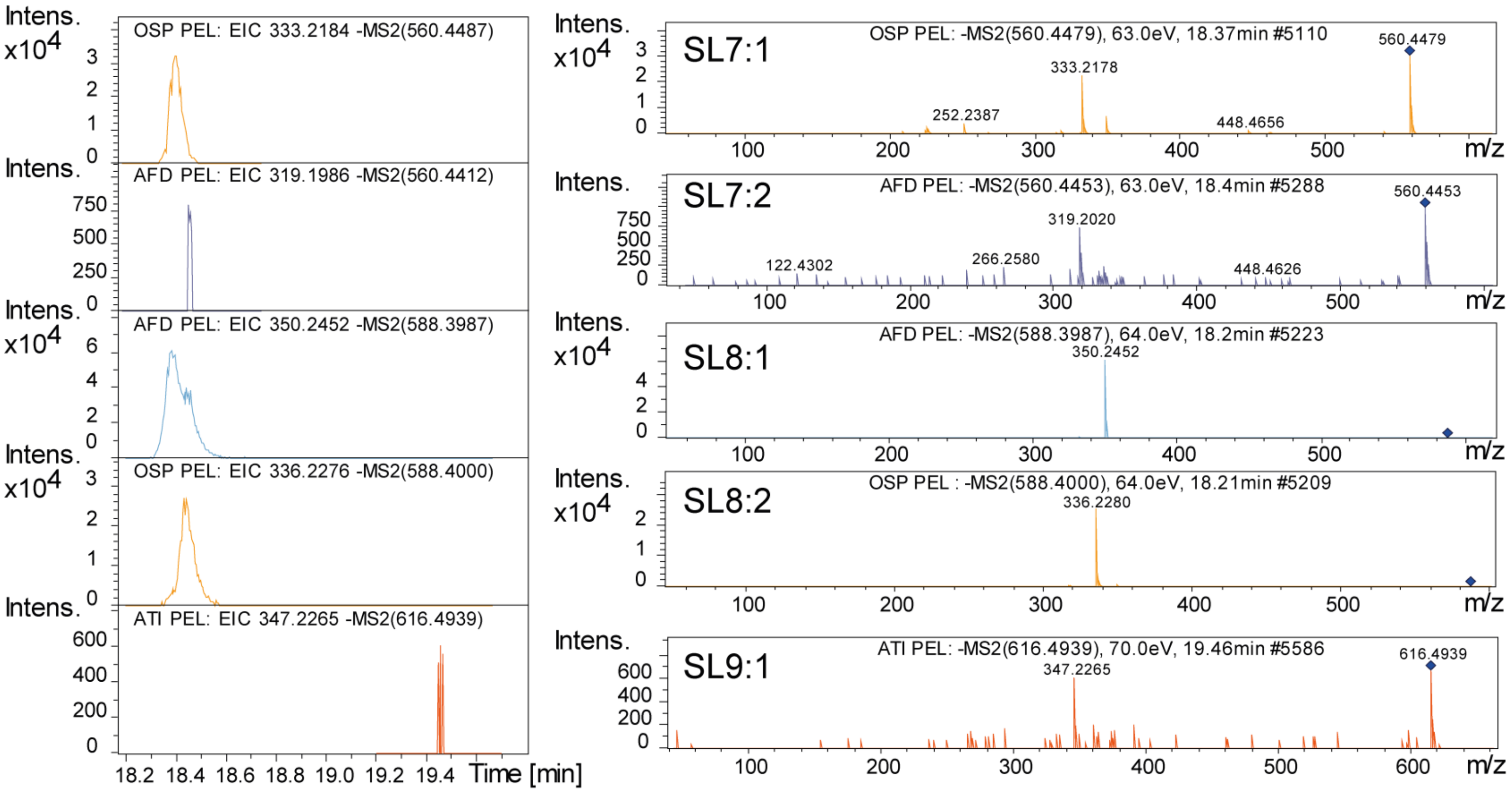

Figure S5.

LC Peak and MS/MS examples of all sixteen identified SLs compounds in bacteria using UHPLC-TOF-MS/MS experiments. Left panel shows extracted ion chromatograms of SL1-SL9 (mass signal values are used from Table 1, with an extraction window of ±0.005 Dalton) measured in methanol extracts of bacterial pellets i.e., OSP, AFD and ATI. Right panel shows corresponding MS/MS data of all sixteen SLs.

Figure S6

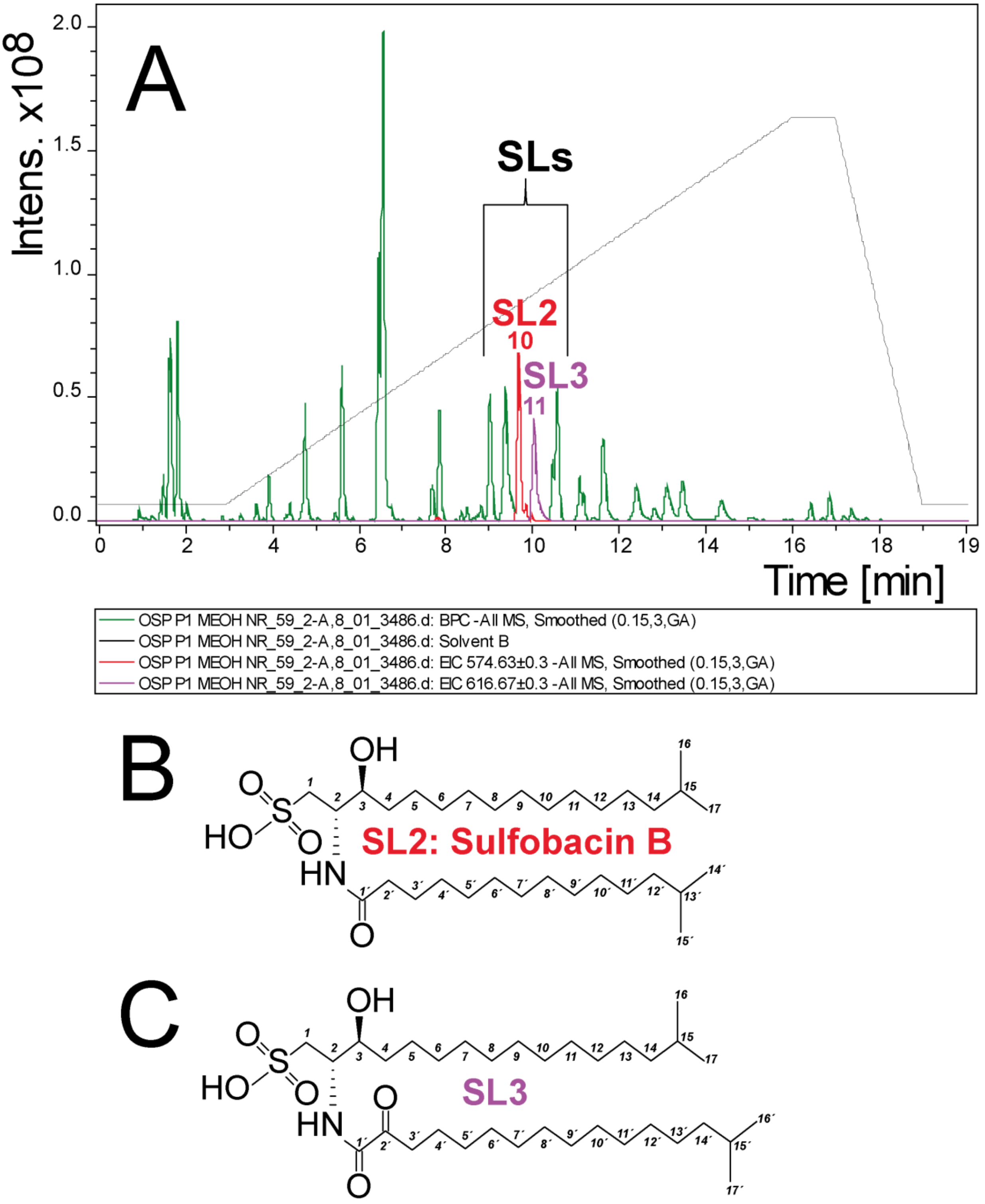

Figure S6.

A: Typical chromatogram of SL2 (fraction 10) and SL3 (fraction 11) as isolated from OSP pellet, using a combination of UHPLC coupled to ion trap mass spectrometer. Insert structures concern SL2 (B) and SL3 (C) compounds as characterised by NMR spectroscopy (see Figs. S7-9 and Tables S5-6).

Figure S7

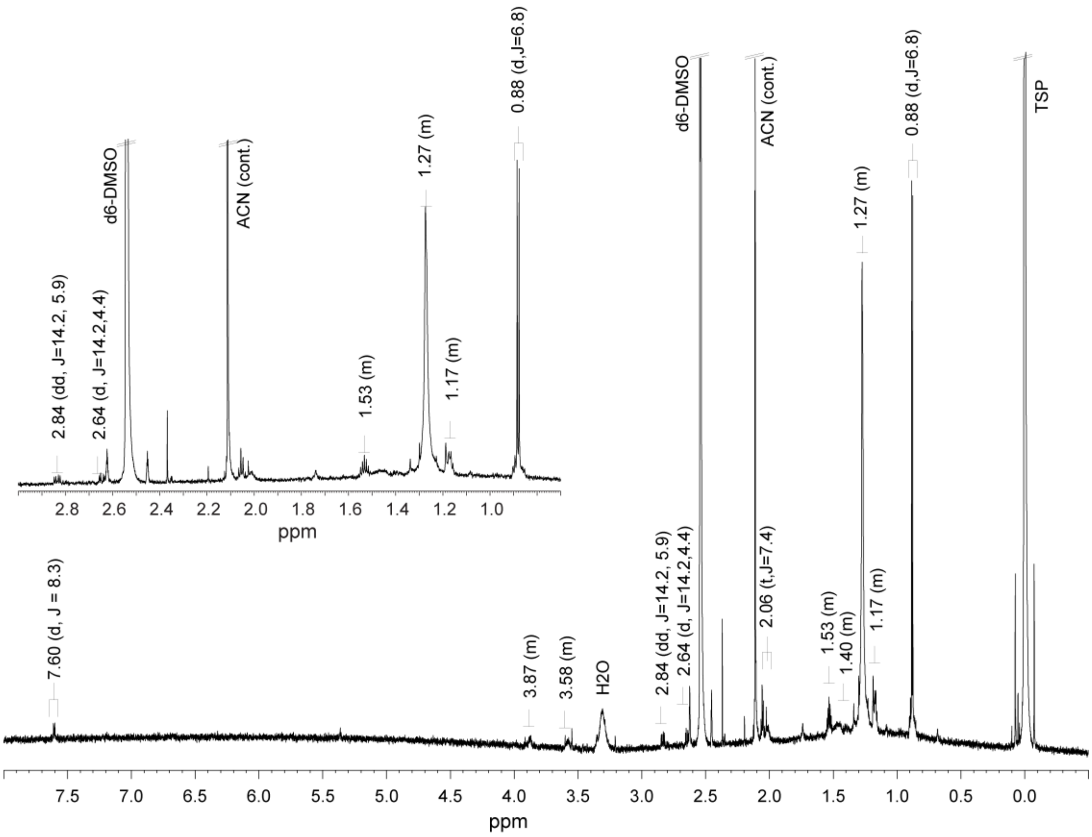

Figure S7.

^{1}H NMR spectrum of SL2 compound (800-MHz; DMSO-d_6, see also Table S5). Corresponding structure of SL2 (Sulfobacin B) is inserted in Fig. S6B.

Figure S8

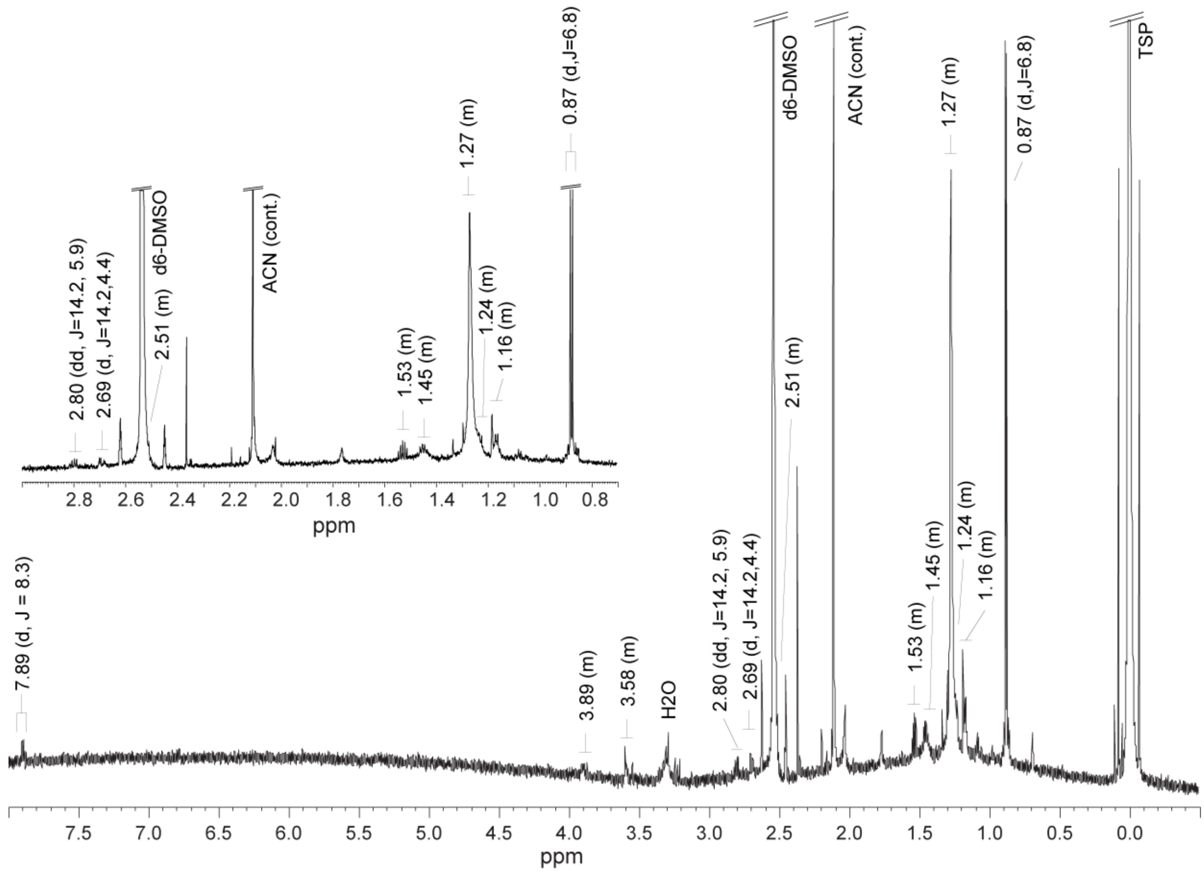

Figure S8.

^{1}H NMR spectrum of SL3 (800 MHz, DMSO-d_6; see also Table S6). Corresponding structure of SL3 is inserted in Fig S6C.

Figure S9

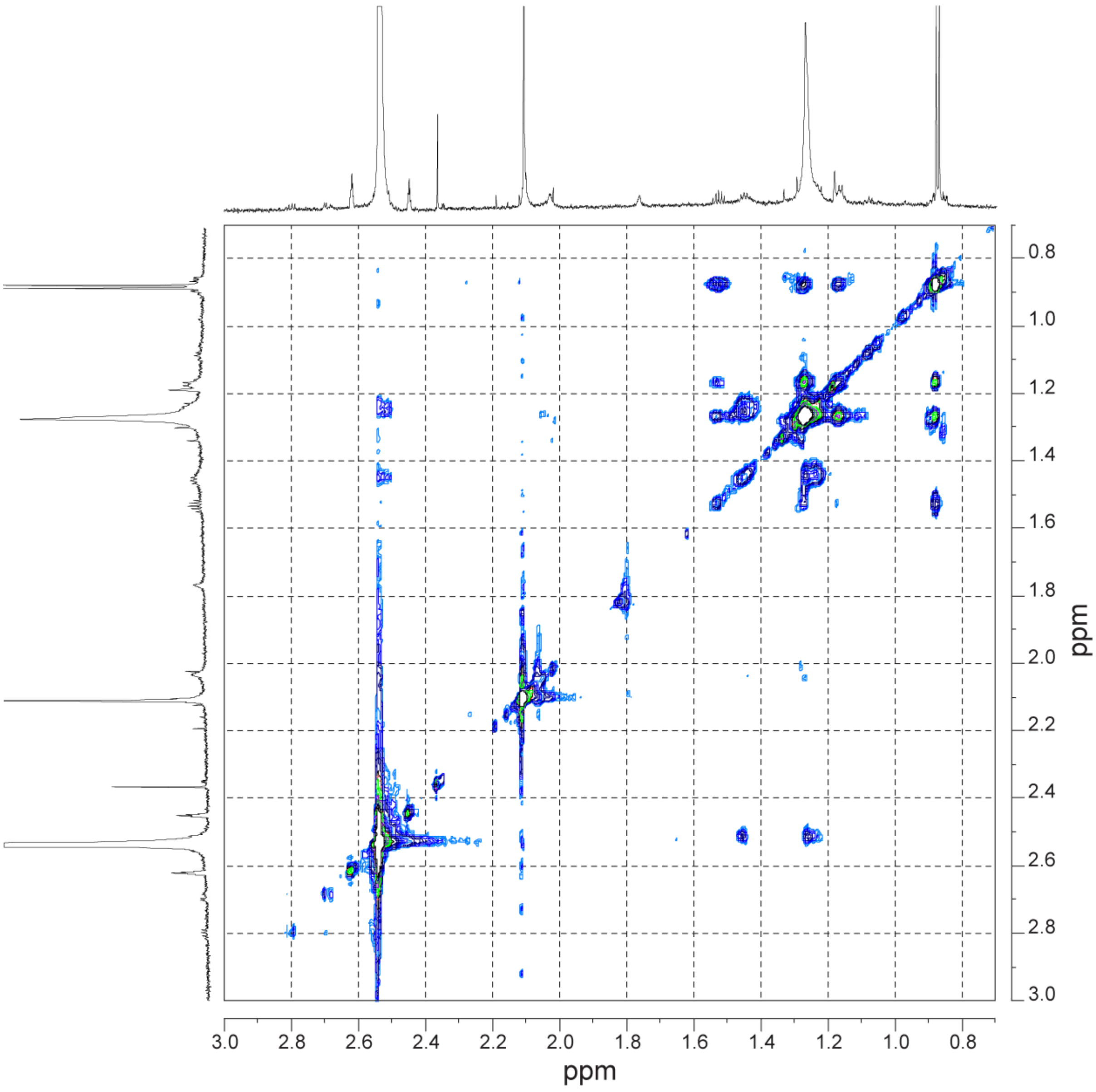

Figure S9.

2D TOCSY NMR spectrum of SL3 compound (800-MHz, DMSO-d_6).

Tables

Table S1.

Overview of putative SLs, detected in cecal samples by means of FT-ICR-MS analysis, including experimental and theoretical mass signal values, molecular formulas, mean intensities, and database (ChemSpider) annotation.

Class	Nr.	Experimental mass signal value [M-H]⁻	error ppm [alignment in 1 ppm]	Theoretical mass signal value [M-H]⁻	Molecular Formula (M)	Mean (Int.)	Detected in n mice total n=7	Found in chemical database[a] (Systematic name, ChemSpider ID)	Structure of SLS[b]
I	SL1	588.46630	-0.71	588.46672	$C_{33}H_{67}O_5N_1S_1$	5.92E+08	7	N-[(E,1S,2R)-2-hydroxy-1-(hydroxymethyl)-5-undecoxy-pent-3-enyl]hexadecane-1-sulfonamide; 26591116	No
I	SL2	574.45070	-0.64	574.45107	$C_{32}H_{65}O_5N_1S_1$	5.16E+08	7	Sulfobacin B; (2R,3R)-3-Hydroxy-15-methyl-2-[(13-methyltetradecanoyl)amino]-1-hexadecanesulfonic acid; 8183902	Yes
II	SL3	616.46118	-0.75	616.46164	$C_{34}H_{67}O_6N_1S_1$	2.28E+08	7	Flavocristamide A; (2R,3R,4E)-3-Hydroxy-2-([(3R)-3-hydroxy-15-methylhexadecanoyl]amino))-15-methyl-4-hexadecene-1-sulfonic acid; 8184566	Yes
I	SL4	602.48236	-0.02	602.48237	$C_{34}H_{69}O_5N_1S_1$	1.86E+08	7	2-[(2-Hydroxyhexadecyl)(palmitoyl)amino]ethanesulfonic acid; 8184566	Yes
II	SL5	602.44598	-0.02	602.44599	$C_{33}H_{65}O_6N_1S_1$	1.71E+08	7	No Hit	
II	SL6	630.47682	-0.75	630.47729	$C_{35}H_{69}O_6N_1S_1$	8.90E+07	7	No Hit	
I	SL7	560.43515	-0.48	560.43542	$C_{31}H_{63}O_5N_1S_1$	6.13E+07	7	No Hit	
II	SL8	588.42997	-0.63	588.43034	$C_{32}H_{63}O_6N_1S_1$	4.04E+07	7	(2S,4E)-3-Hydroxy-2-[(2-hydroxy-13-methyltetradecanoyl)amino]-15-methyl-4-hexadecene-1-sulfonic acid; 10477903	Yes
I	SL9	616.49753	-0.79	616.49802	$C_{35}H_{71}O_5N_1S_1$	1.95E+07	7	No Hit	
I	SL10	586.45069	-0.65	586.45107	$C_{33}H_{65}O_5N_1S_1$	1.89E+07	7	No Hit	
I	SL11	572.43537	-0.09	572.43542	$C_{32}H_{63}O_5N_1S_1$	1.22E+07	7	2R,3R,4E)-3-Hydroxy-2-(tetradecanoylamino)-4-octadecene-1-sulfonic acid; 24823201	Yes
I	SL12	600.46670	-0.03	600.46672	$C_{34}H_{67}O_5N_1S_1$	1.12E+07	7		yes
II	SL13	618.47694	-0.57	618.47729	$C_{34}H_{69}O_6N_1S_1$	5.67E+06	7	Sulfobacin A; (2R,3R,4E)-3-Hydroxy-2-(palmitoylamino)-4-octadecene-1-sulfonic acid; 24823202	Yes
II	SL14	656.49262	-0.49	656.49294	$C_{37}H_{71}O_6N_1S_1$	4.98E+06	7	(2S,3R,4E,8E)-3-Hydroxy-2-[methyl(stearoyl)amino]-4,8-octadecadien-1-yl hydrogen sulfate; 8898327	No
II	SL15	614.44563	-0.59	614.44599	$C_{34}H_{65}O_6N_1S_1$	4.31E+06	7	No Hit	
II	SL16	644.49278	-0.25	644.49294	$C_{36}H_{71}O_6N_1S_1$	4.30E+06	7	No Hit	
II	SL17	600.43034	0.00	600.43034	$C_{33}H_{63}O_6N_1S_1$	2.85E+06	6	No Hit	
II	SL18	628.46117	-0.75	628.46164	$C_{35}H_{67}O_6N_1S_1$	2.30E+06	5	No Hit	

a: Search performed by means of molecular formula in ChemSpider and PubChem

b: Structures found in databases were compared to the already reported known structures of sulfobacin B, A and Flavocristamide A[7]

Table S2.

Summary of all eighteen SLs with their measured retention time (RT), theoretical mass signal values and molecular formulas of parent and fragment ions and applied collision energies in eV. MS/MS were performed in negative electrospray ionization mode. This table also represents information about major parent-fragment ions that were used for all MS/MS experiments that are highlighted in Figure 3.

	SL	RT in min	Theoretical mass signal value of parent ion	MF of Parent Ion	Theoretical mass signal value of fragment ion	MF of Fragment Ion	Collision energy (eV)
1	SL1:1	19	588.466719	$C_{33}H_{66}NO_5S^-$	347.2261	$C_{18}H_{35}O_4S^-$	70
2	SL1:2	19	588.466719	$C_{33}H_{66}NO_5S^-$	333.2105	$C_{17}H_{33}O_4S^-$	70
3	SL2	18.6	574.451069	$C_{32}H_{64}NO_5S^-$	333.2105	$C_{17}H_{33}O_4S^-$	70
4	SL3:2	18.8	616.461633	$C_{34}H_{66}NO_6S^-$	350.2371	$C_{17}H_{36}NO_4S^-$	70
5	SL3:1	18.8	616.461633	$C_{34}H_{66}NO_6S^-$	364.2527	$C_{18}H_{38}NO_4S^-$	70
6	SL4:1	19.2	602.482369	$C_{34}H_{68}NO_5S^-$	347.2261	$C_{18}H_{35}O_4S^-$	70
7	SL4:2	19.2	602.482369	$C_{34}H_{68}NO_5S^-$	333.2105	$C_{17}H_{33}O_4S^-$	70
8	SL5:1	18.5	602.445983	$C_{33}H_{64}NO_6S^-$	350.2371	$C_{17}H_{36}NO_4S^-$	70
9	SL5:2	18.5	602.445983	$C_{33}H_{64}NO_6S^-$	364.2527	$C_{18}H_{38}NO_4S^-$	70
10	SL5:3	18.5	602.445983	$C_{33}H_{64}NO_6S^-$	336.2214	$C_{16}H_{34}NO_4S^-$	70
11	SL6:1	19.1	630.477283	$C_{35}H_{68}NO_6S^-$	378.2683	$C_{19}H_{40}NO_4S^-$	70
12	SL6:2	19.1	630.477283	$C_{35}H_{68}NO_6S^-$	364.2527	$C_{18}H_{39}NO_4S^-$	70
13	SL7:1	18.4	560.435419	$C_{31}H_{62}NO_5S^-$	333.2105	$C_{17}H_{33}O_4S^-$	63
14	SL7:2	18.4	560.435419	$C_{31}H_{62}NO_5S^-$	319.1948	$C_{16}H_{31}O_4S^-$	63
15	SL8:1	18.2	588.430333	$C_{32}H_{62}NO_6S^-$	350.2371	$C_{17}H_{36}NO_4S^-$	64
16	SL8:2	18.2	588.430333	$C_{32}H_{62}NO_6S^-$	336.2214	$C_{16}H_{34}NO_4S^-$	64
17	SL9:2	19.6	616.498019	$C_{35}H_{70}NO_5S^-$	347.2261	$C_{18}H_{35}O_4S^-$	70
18	SL9:1	19.6	616.498019	$C_{35}H_{70}NO_5S^-$	361.2418	$C_{19}H_{37}O_4S^-$	70

MF = molecular formula; RT = retention time;
eV = electron volt

Table S3.

Body weights of SAFF (n=9), LARD (n=10) and NC (n=7) fed mice before and after dietary intervention of 3 weeks.

Body weight in g	Start	End (3 weeks)
SAFF 1	26.3	31.2
SAFF 2	27	30.6
SAFF 3	27.2	32.1
SAFF 4	31.5	41.4
SAFF 5	26	31
SAFF 6	27.8	33.4
SAFF 7	31.9	38.6
SAFF 8	41.4	43
SAFF 9	30.1	35
LARD 1	29.2	38.7
LARD 2	26.8	34
LARD 3	26	32.1
LARD 4	27.9	34.8
LARD 5	25.5	30.8
LARD 6	30	40
LARD 7	30.4	40
LARD 8	27.2	33.6
LARD 9	27.7	30.5
LARD 10	31	37
NC 1	35.6	35.3
NC 2	29.3	29.9
NC 3	29.1	28.8
NC 4	30	29.8
NC 5	27	30.8
NC 6	26.1	26.5
NC 7	32.7	34.1
P-value (Welch t-test); SAFF vs. NC	0.977	0.041
P-value (Welch t-test); LARD vs. NC	0.223	0.016

Table S4.

Summary of all SLs compounds detected in pure cultures of different strains together with C17-Capnine precursor with 1 = detected or 0 = not detected.

	AFD	OSP	APU	ASH	FJO	CGL	AIN	AON	ATI	AOB	AIH	Number of Bacteria
SL1:1	1	0	0	1	1	0	1	1	1	1	0	7
SL1:2	0	1	1	0	1	0	1	1	1	1	1	8
SL2	1	1	1	1	1	1	1	1	1	1	1	11
SL3:1	1	1	0	1	0	0	0	1	1	1	0	6
SL3:2	1	1	1	1	0	1	1	1	1	1	1	10
SL4:2	1	1	1	1	1	0	1	1	1	1	1	10
SL5:1	1	1	1	1	1	1	1	1	1	1	1	11
SL5:2	1	0	0	1	1	0	0	1	1	1	0	6
SL5:3	1	1	1	1	1	1	1	1	1	1	0	10
SL6:1	0	0	0	0	0	0	0	0	1	0	0	1
SL6:2	1	1	1	1	1	1	1	1	1	1	0	10
SL7:1	0	1	1	1	1	0	1	1	1	1	1	9
SL7:2	1	1	1	1	1	0	1	1	1	1	1	10
SL8:1	1	0	0	1	0	0	0	0	1	0	0	3
SL8:2	0	1	0	0	1	0	1	0	1	0	0	4
SL9:1	0	0	0	0	0	0	0	1	1	0	0	2
**Number of detected SLs**	11	11	9	12	11	5	11	13	16	12	7	
**Precursor:C17-Capnine**	1	1	1	1	1	1	1	1	1	1	1	

Alistipes finegoldii DSM17242 (AFD); *Odoribacter splanchnicus* DSM 20712 (OSP), *Alistipes putredinis* DSM 17216 (APU); *Alistipes shahii* DSM 19121 (ASH); *Flavobacterium johnsoniae* DSM 2064 (FJO); *Chryseobacterium gleum* DSM 16776 (CGL); *Alistipes indistinctus* DSM 22520 (AIN); *Alistipes onderdonkii* DSM 19147 (AON), *Alistipes timonensis* DSM 25383 (ATI), *Alistipes* obesi DSM 25724 (AOB) and *Alistipes ihumii* DSM 26107 (AIH).

Table S5.

^{1}H NMR data for Sulfobacin B (fraction 10) in DMSO-d_6 at 800-MHz (see also Figure S7).

**Position**	**δH in ppm (multiplicities, coupling constants in Hz)**
1	2.64 (dd, J=14.2, 4.4)
	2.84 (dd, J=14.2, 5.9)
2	3.87 (m)
3	3.58 (m)
4-13	1.27 (m)
14	1.17 (m)
15	1.53 (m)
16	0.88 (d, J=6.8)
2-NH	7.60 (d, J=8.3)
3-OH	4.86*
2'	2.06 (t, J=7.4)
3'	1.40 (m)
4'-11'	1.27 (m)
12'	1.17 (m)
13'	1.53 (m)
14'	0.88 (d, J=6.8)

* Not visible in the ^{1}H spectrum, only in 2D-TOCSY.

Table S6.

^{1}H NMR data for SL3 (fraction 11) in DMSO-d_6 at 800-MHz (see also Figure S8).

**Position**	**δH in ppm (multiplicities, coupling constants in Hz), measured**	**δH in ppm (multiplicities), predicted in ACD/Labs**
1	2.69 (dd, J=14.2, 4.4)	2.65 (dd)
	2.80 (dd J=14.2, 5.9)	2.75 (dd)
2	3.89 (m)	4.06 (m)
3	3.58 (m)	3.50 (m)
4-6	1.53 (m)	1.35-1.55 (m)
7-13	1.27 (m)	1.27 (m)
14	1.16 (m)	1.13 (m)
15	1.53 (m)	1.51 (m)
16	0.87 (d, J=6.8)	0.87 (d)
2-NH	7.89 (d, J=8.3)	7.22 (d)
3-OH	4.82*	(5.80)
3'	2.51 (m)	2.65 (m)
4'	1.45 (m)	1.62 (m)
5'	1.24 (m)	1.35 (m)
6'-13'	1.27 (m)	1.27 (m)
14'	1.16 (m)	1.13 (m)
15'	1.53 (m)	1.51 (m)
16'	0.87 (d, J=6.8)	0.87 (d)

* Not visible in the ^{1}H spectrum, only in 2D-TOCSY.

Table S7.

Arithmetic mean for analyzed SL1-SL9 (normalized peak areas (weight of wet cecal content)) in GF, SPF and Alistipes mice.

SL Nr.	Mean GF	Mean SPF	Mean Alistipes
SL1:1	0	446.008798	4224.69536
SL1:2	0	480.669941	446.716454
SL2	0	666.406952	6118.54361
SL3:1	0	1471.56781	411.857244
SL3:2	0	2218.19506	12324.9744
SL4:1	0	183.814361	592.455535
SL4:2	0	7.00370882	593.95655
SL5:1	0	2997.22355	911.291959
SL5:2	0	153.66799	1276.90871
SL5:3	0	143.480412	0
SL6:1	0	1.48031125	50.319415
SL6:2	0	1578.5011	7670.61542
SL7:1	0	31.4454543	0
SL7:2	0	17.0296793	1.73662738
SL8:1	0	525.1622	2617.179
SL8:2	0	556.428785	0
SL9:1	0	0	310.900452
SL9:2	0	0	15.9851831
SL1:1/SL2	0	0.66927393	0.69047401
SL1:2/SL2	0	0.7212859	0.07301026

Supplementary references

1 Kamiyama, T. *et al.* Sulfobacins A and B, novel von Willebrand factor receptor antagonists. I. Production, isolation, characterization and biological activities. *J Antibiot (Tokyo)* **48**, 924-928, (1995).

2 Schmieder, R. & Edwards, R. Quality control and preprocessing of metagenomic datasets. *Bioinformatics* **27**, 863-864, (2011).

3 Altschul, S. F., Gish, W., Miller, W., Myers, E. W. & Lipman, D. J. Basic local alignment search tool. *J Mol Biol* **215**, 403-410, (1990).

4 Camacho, C. *et al.* BLAST+: architecture and applications. *BMC Bioinformatics* **10**, 421, (2009).

5 Kanehisa, M. & Goto, S. KEGG: kyoto encyclopedia of genes and genomes. *Nucleic Acids Res* **28**, 27-30, (2000).

6 Huson, D. H., Auch, A. F., Qi, J. & Schuster, S. C. MEGAN analysis of metagenomic data. *Genome Res* **17**, 377-386, (2007).

7 Takikawa, H., Nozawa, D., Kayo, A., Muto, S.-e. & Mori, K. Synthesis of sphingosine relatives. Part 22. Synthesis of sulfobacin A, B and flavocristamide A, new sulfonolipids isolated from Chryseobacterium sp. *Journal of the Chemical Society, Perkin Transactions 1*, 2467-2477, (1999).
